# Simulation tool for assessing the release and environmental distribution of nanomaterials

**DOI:** 10.3762/bjnano.6.97

**Published:** 2015-04-13

**Authors:** Haoyang Haven Liu, Muhammad Bilal, Anastasiya Lazareva, Arturo Keller, Yoram Cohen

**Affiliations:** 1 Center for the Environmental Implications of Nanotechnology, California NanoSystems Institute, University of California, Los Angeles, CA 90095, USA; 2 Chemical and Biomolecular Engineering Department, University of California, Los Angeles, Los Angeles, CA 90095, USA,; 3 Bren School of Environmental Science & Management, University of California, Santa Barbara, Santa Barbara, CA 91306, USA

**Keywords:** engineered nanomaterials, environmental exposure assessment, life cycle assessment, nanoinformatics, web-based simulation tool

## Abstract

An integrated simulation tool was developed for assessing the potential release and environmental distribution of nanomaterials (RedNano) based on a life cycle assessment approach and multimedia compartmental modeling coupled with mechanistic intermedia transport processes. The RedNano simulation tool and its web-based software implementation enables rapid “what-if?” scenario analysis, in order to assess the response of an environmental system to various release scenarios of engineered nanomaterials (ENMs). It also allows for the investigation of the impact of geographical and meteorological parameters on ENM distribution in the environment, comparison of the impact of ENM production and potential releases on different regions, and estimation of source release rates based on monitored ENM concentrations. Moreover, the RedNano simulation tool is suitable for research, academic, and regulatory purposes. Specifically, it has been used in environmental multimedia impact assessment courses at both the undergraduate and graduate levels. The RedNano simulation tool can also serve as a decision support tool to rapidly and critically assess the potential environmental implications of ENMs and thus ensure that nanotechnology is developed in a productive and environmentally responsible manner.

## Introduction

Engineered nanomaterials (ENMs) are reported to be utilized in more than 1,000 commercial products owing to their unique size-related beneficial properties [1–4]. It is estimated that global ENM production levels will be in excess of 340,000 tons by 2016 [5]. Given the rapid growth of nanotechnology, it is critical to assess the potential impacts associated with ENMs and thus to ensure that nanotechnology is developed in an environmentally compatible manner. In this regard, various environmental impact assessment (EIA) frameworks have been proposed [6], which all require knowledge of the potential environmental distribution of ENMs in addition to their potential toxicological effects. However, reported ENM source release rates, environmental monitoring data of ENM concentrations, as well as suitable ENM measurement techniques are presently scarce. Thus, computational models have been proposed as support tools to estimate ENM release rates [7–8] and potential environmental exposure concentrations [9–11].

It has been proposed that analysis of the multimedia environmental distribution and exposure concentrations of contaminants can be accomplished via a tiered approach [12]. A screening level assessment (tier-1 analysis) can be carried out based on multimedia compartmental models (MCMs) [12] to identify major exposure pathways and to monitor data gaps. In such analysis, the environmental entry, movement, and distribution of contaminants are described by a set of mathematical expressions. Specifically, MCMs require mechanistic quantification of intermedia transport rates (e.g., dry and wet deposition, sedimentation, dissolution) and rates of contaminant release to various environmental media. Typically, such a screening level analysis is expected to provide an order of magnitude (or better) assessment. Although MCMs have been developed to estimate non-steady-state (i.e., temporal dynamic) environmental concentrations of gaseous and dissolved chemical pollutants (e.g., Mend-Tox [13–14], CalTOX [15], TRIM.FaTE [16]), these are not directly applicable for ENMs. Unlike gaseous and dissolved chemical pollutants, for which interphase mass transport rates are governed by chemical potential (fugacity) driving forces that are constrained by thermodynamic equilibrium, the intermedia transport of ENMs is governed by physical transport processes of particulate matter. Therefore, a description of the environmental fate and transport of ENMs requires the particle size distribution (PSD) to be accounted for within the modeling framework, as well as the PSD dependence of the various transport processes. Higher tier analyses, which may include the use of detailed single medium models, can provide higher spatial resolution of the predicted ENM distribution for the studied region (in contrast to a regional average of ENM media concentrations). However, such an approach requires extensive site-specific geographical information and meteorological data for the target region (i.e., 
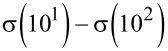
 higher relative to the tier-1 approach [14]), and thus can be more complex and computationally demanding.

Irrespective of model complexity, an important factor in assessing the environmental multimedia distribution of ENMs is their release rates. In order to estimate ENMs release rates, life cycle inventory assessment (LCIA) based approaches have been developed to track the target ENM mass throughout its life cycle from production, through use, to final disposal and/or release into the environment. LCIA approaches are based on ENM production rates and empirical transfer coefficients that quantify the fraction of mass transferred between compartments (including technical compartments, such as waste processing facilities, as well as environmental compartments, such as air, water and soil) [7–8,17–19]. Although there are uncertainties in the LCIA approaches (primarily due to the inherent uncertainty in the estimated ENM production rates and intercompartmental transfer coefficients [7]), such methods are considered at present as being reasonably suitable for assessing potential ENM release rates [7,17]. There have also been attempts to extend LCIA-based methods to estimate the ENM media concentrations (e.g., via material flow analysis) [17–19] relying on empirically estimated media transfer coefficients under laboratory (i.e., not environmental) conditions. In the above methodology, estimated transport rates may violate constraints imposed by intermedia transport mechanisms [9]. A recently proposed approximate treatment for steady-state ENM multimedia concentrations was provided by SimpleBox4nano [11], which is yet to be validated against environmentally measured concentrations of particulate matter. This model considers a range of intermedia transport processes (including episodic events such as rain scavenging) as continuous processes, with constant rate coefficients throughout the simulation period. SimpleBox4nano also does not consider temporal variability of meteorological conditions or source releases, and processes such as wind resuspension, aerosolization, foliage washoff, and uptake by biological organisms are not included. It is stressed that SimpleBox4nano only considers the average particle size in each particle class (primary ENM (with size of 10 nm), ENM attached to colloids, and ENM attached to larger particles), while assuming an arbitrary value of 0.1 for both aggregation and attachment efficiencies [11]. As a consequence, the above approach does not account for the temporal dynamics of multimedia distribution and the strong dependence of ENM intermedia transport on the complete PSDs [9].

In earlier work, a multimedia environmental distribution of nanomaterials (MendNano) model was developed [9] based on a mechanistic description of various intermedia transport and reaction (including dissolution) processes, which considers the complete PSD of ENMs and ambient particulates. This study reported that dry and wet depositions (from air) are important intermedia transport pathways for ENM removal from the atmosphere and their input to the aquatic and terrestrial environments, the latter being particularly significant in the absence of direct ENM release to those compartments. Also, the dissolution of sparingly soluble ENMs in the water compartment can be the dominant mechanism for removal of particulate ENMs from water. MendNano was also applied to the modeling of the environmental distribution of semi-volatile organics. These organics adsorb onto ambient particles [20–21] and thus their transport behavior is governed by the particle phase as is the case with ENMs [9,12]. Simulation results have demonstrated excellent agreement with environmental monitoring data to within a factor of 2 or better [9], which is an acceptable level for compartmental models [22–24].

Compartmental models can be used to provide a first-tier analysis for estimating the magnitudes of potential ENM exposure concentrations. However, in order to support timely decision analysis regarding the potential environmental impact of ENMs, it is imperative to make available integrated tools that enable rapid analysis. Accordingly, in the present work, an integrated simulation tool for estimating the potential release and the environmental distribution of nanomaterials (RedNano) was developed. This tool integrates MendNano [9] with a LCIA-based model for estimating ENM release rates [7,25]. RedNano is a simulation tool suitable for estimating the potential environmental ENM release and distribution, for performing multimedia scenario analysis, and for evaluating the significance of intermedia transport pathways. RedNano has been deployed as a web application and was developed as a modular system. Its structure and utility are demonstrated in the present study with a number of illustrative use cases.

## Computational Modeling

### Overview of RedNano simulation tool

**Figure 1:**
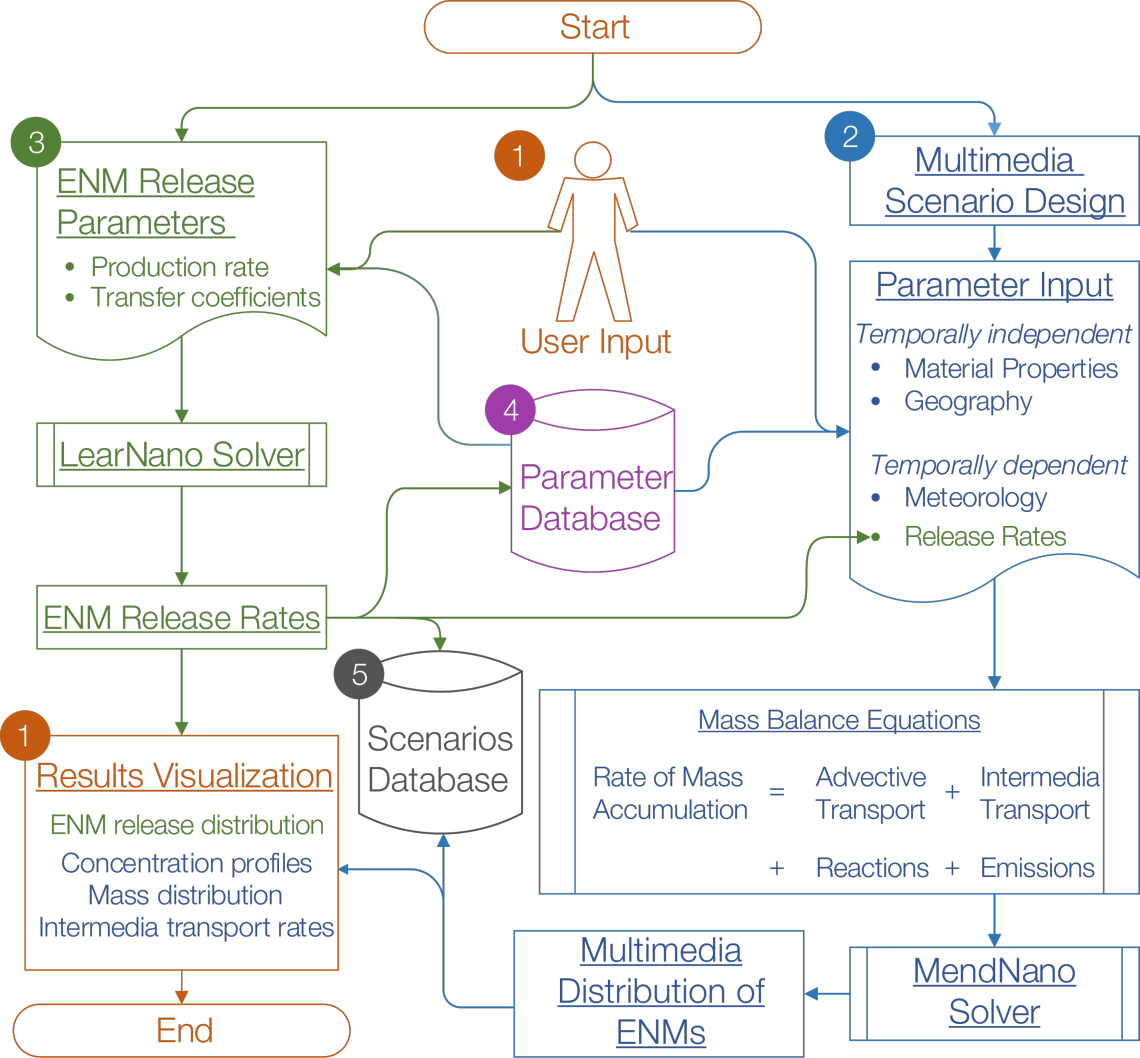
Overview of the release and environmental distribution of nanomaterials (RedNano) simulation tool and its components: (1) GUI, (2) MendNano, (3) LearNano, (4) parameter database, and (5) scenarios database.

RedNano consists of five main elements (Figure 1): (1) user interface for scenario design and results visualization, (2) MendNano, which is a fate and transport model for estimating environmental ENM concentrations, (3) lifecycle environmental assessment for release of nanomaterials (LearNano) model for estimating ENM release rates, (4) a parameter database, and (5) a repository for building a library of scenarios and simulation cases. The RedNano graphical user interface (GUI) provides guidance for scenario design and parameter specification; the latter may be obtained from an integrated parameter database, input manually, or calculated by various submodels. Based on the designed scenario, MendNano computes the multimedia mass distribution of ENMs given a release rate and/or initial concentration of the selected ENMs in one or more of the environmental compartments. Simulation results are then graphically represented via visualization modules as well as provided in standard numerical formats. Additionally, scenario input data as well as intermediary and final simulation results are stored in the scenario database. The RedNano integrated simulation tool was designed as a client–server web application using a standard web development environment (i.e., HTML, PHP, JavaScript, MySQL).

### MendNano

**Figure 2:**
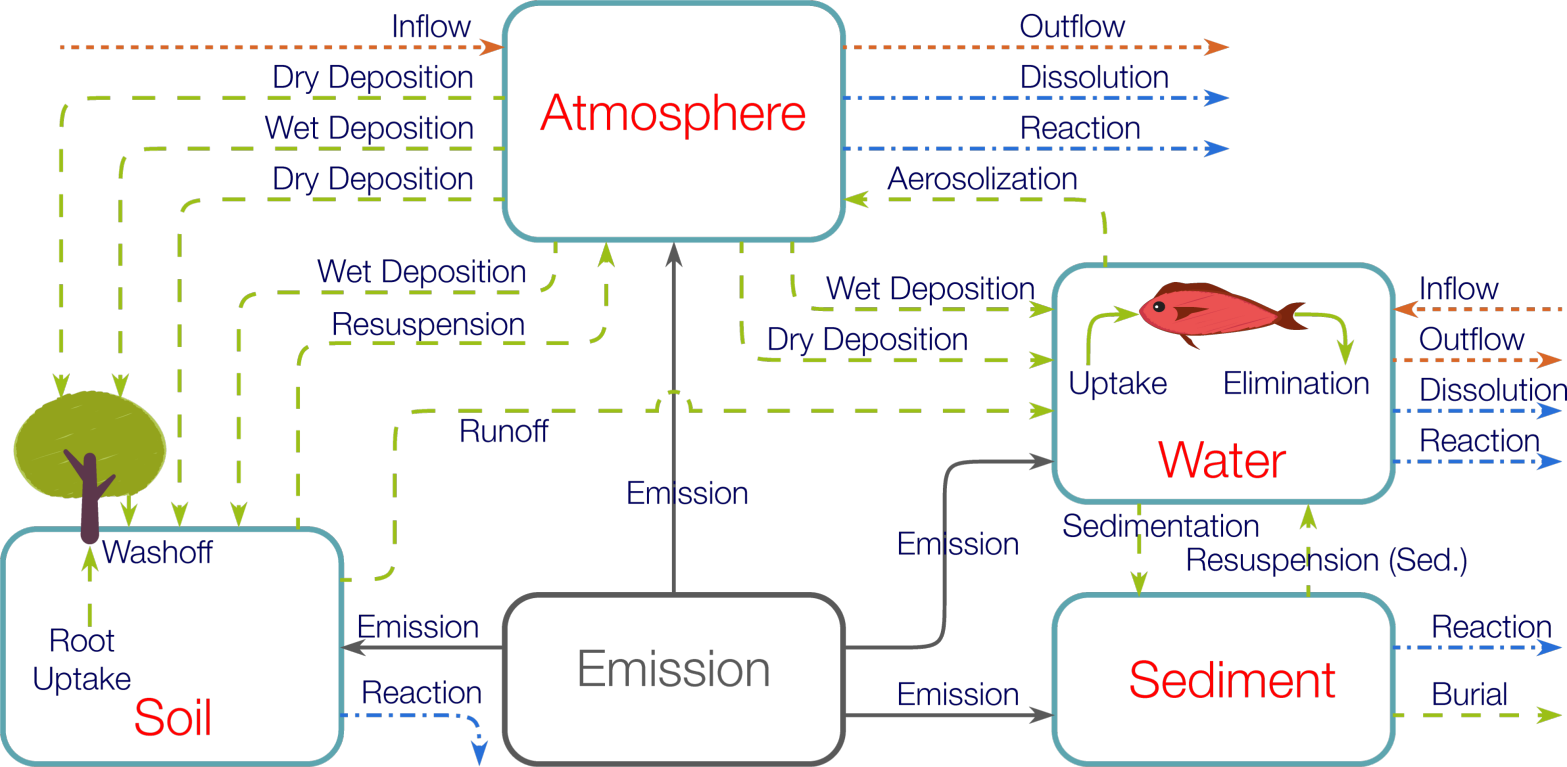
Transport processes in MendNano. Green dashed lines represent intermedia transport processes, blue dash-dot lines represent reactions (including dissolution) within the compartments that eliminate the ENM from particle phase, orange dotted lines represent advection (i.e., transport of ENMs via the flow of air and water) into and out of the given compartment, and gray solid lines represent emissions (i.e., ENM release events into the compartments).

The theoretical basis describing the dynamic distribution of ENMs in the multimedia environment is provided in detail elsewhere [9]. Briefly, MendNano treats the multimedia environment as a set of well-mixed compartments (e.g., air, water, soil, sediment, biotas) linked via intermedia transport processes (ITP) meaning among compartments (e.g., dry/wet deposition, resuspension, sedimentation, dissolution) as listed in Figure 2. The resulting unsteady state, mass balance, ordinary differential equations (Supporting Information File 1, Equation S1) are then solved to obtain the mass of the ENMs in the various environmental compartments, and thus the temporal evolution of their mass distribution, concentration, and intermedia transport rate. Intermedia transport rates are specified by mechanistic transport processes, and are governed by geographical and meteorological parameters, as well as material properties. The compartmental modeling approach, which is generally suitable for regional assessments [26–28] of a minimum area of 1 km^2^ [12], lends itself to screening level analysis. Spatial resolution, however, may be increased by using nested or subcompartments, as well as via hybrid approaches that integrate spatial and well-mixed compartments [14]. In addition, the simulation time should be greater than the longest convective residence time in the model compartments (e.g., hours to days for air and water, respectively [12]). MendNano accounts for the complete PSD of both ENMs and ambient particulates by discretizing the PSD into bins, and the association of ENMs with ambient particulates is described by an attachment factor [9]. The PSD of ambient particulates is typically taken to be self-preserving [29–33], but may be altered when there is significant removal (e.g., during precipitation events). The PSD of ENMs may also be altered in a given compartment as the result of intermedia transport processes such as dry and wet deposition from the atmosphere, gravitational settling in aqueous systems, as well as dissolution and reaction processes in air and water (Figure 2).

MendNano includes modules for: (a) mechanistic submodels for rates of intermedia transport processes [9,12], (b) dynamic compartmental mass balance equations consisting of a set of 50–204 (depending on the user-specified scenario) ordinary differential equations (ODEs), (c) event tracking (for episodic events, e.g., precipitation, wind resuspension), and (d) an ODE solver. The modular construction of MendNano allows for adding/upgrading compartments and transport submodels as new information becomes available (e.g., biological compartments and associated uptake mechanisms). The compartmental mass balance ODEs (Supporting Information File 1, Equation S1) are solved via the Adams–Bashforth–Moulton predictor–corrector method [34], with time steps dynamically selected to achieve the numerical solution error (in terms of compartmental ENM mass) set with 0.1% relative error tolerance (defined as percent change in two consecutive solutions). At each time step, the rates of advective (i.e., via air and water flow) and intermedia transport, reactions, and source release are computed based on the temporally varying parameters (e.g., wind speed, temperature, biological organism mass, ENM release rates).

### LearNano

**Figure 3:**
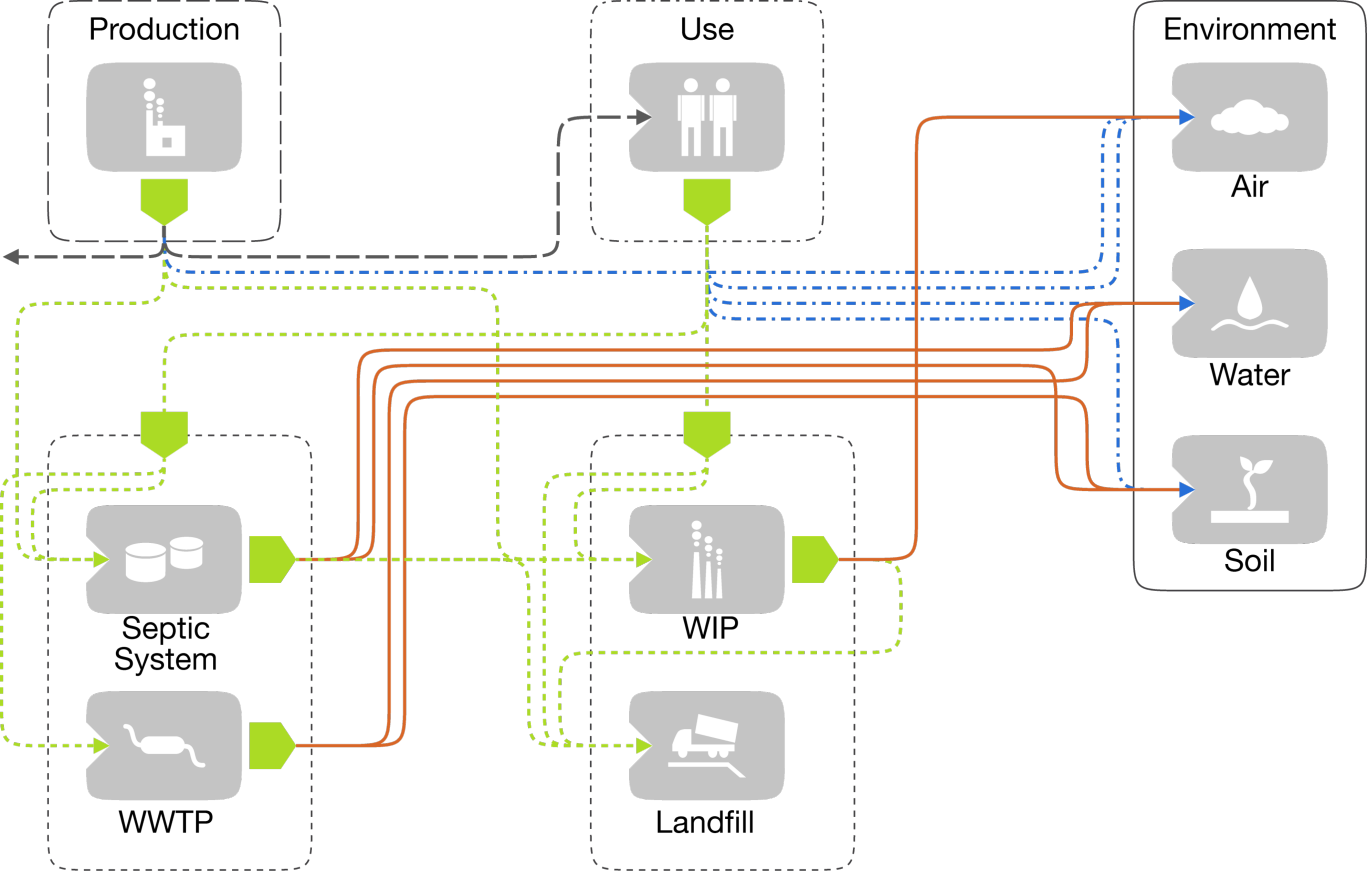
Lifecycle tracking of ENMs. The various lines represent the paths for which transfer coefficients quantify the portion of ENMs transferred from the source to the target compartments. Blue dash-dot lines represent direct release to environmental compartments from production and use, green dotted lines represent ENM transfer from production and use to waste processing facilities, orange solid lines represent indirect release to environmental compartments from waste processing facilities, and gray dashed lines represent import/export and ENM transfer from production to phase.

Estimation of the ENM release rates can be accomplished by the LCIA modeling approach as described in detail elsewhere [7,17]. Briefly, in LCIA-based models, reported ENM mass production rates [5] are allocated to the various ENM applications (e.g., paints, cosmetics, electronics, catalysts), waste processing facilities (i.e., technical compartments), and eventually environmental compartments (Figure 3) [7,17]. Transfer coefficients, which are dependent on the ENM type, ENM application, and region under consideration [7,17], then serve to quantify the fraction of ENMs entering the “source” compartments that are subsequently transferred to the “target” compartment (Figure 3). Accordingly, a series of algebraic mass balance equations that describe ENM mass release rates related to the various environmental compartments [7,17] are incorporated in LearNano (Supporting Information File 1, Equations S2–S4).

**Figure 4:**
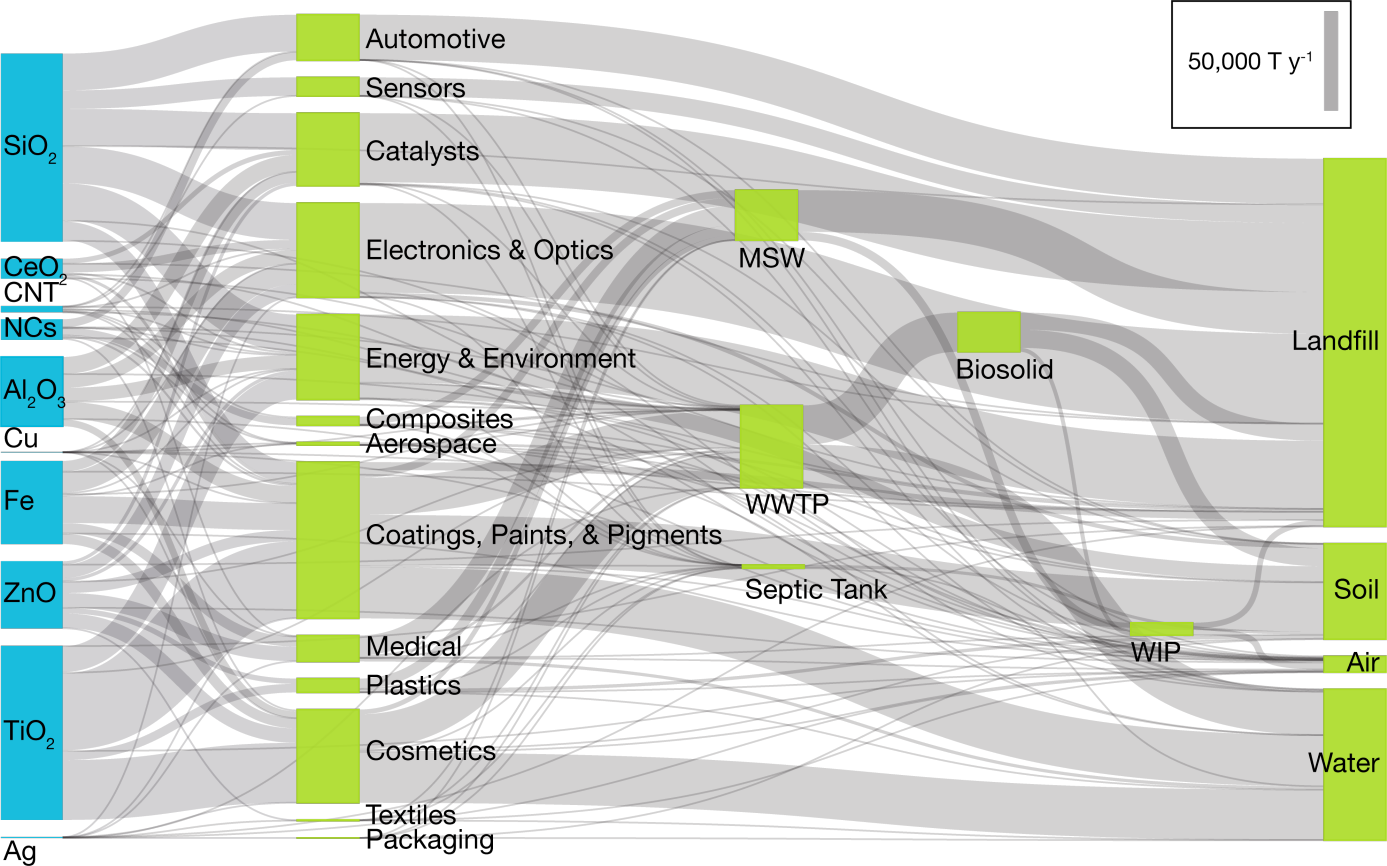
Sankey diagram depicting the flows of different ENMs from production and use, through technical compartments, to disposal and release to the environment. The vertical size of the bars and thickness of the links represent the magnitude of the ENM mass transfer rate.

**Figure 5:**
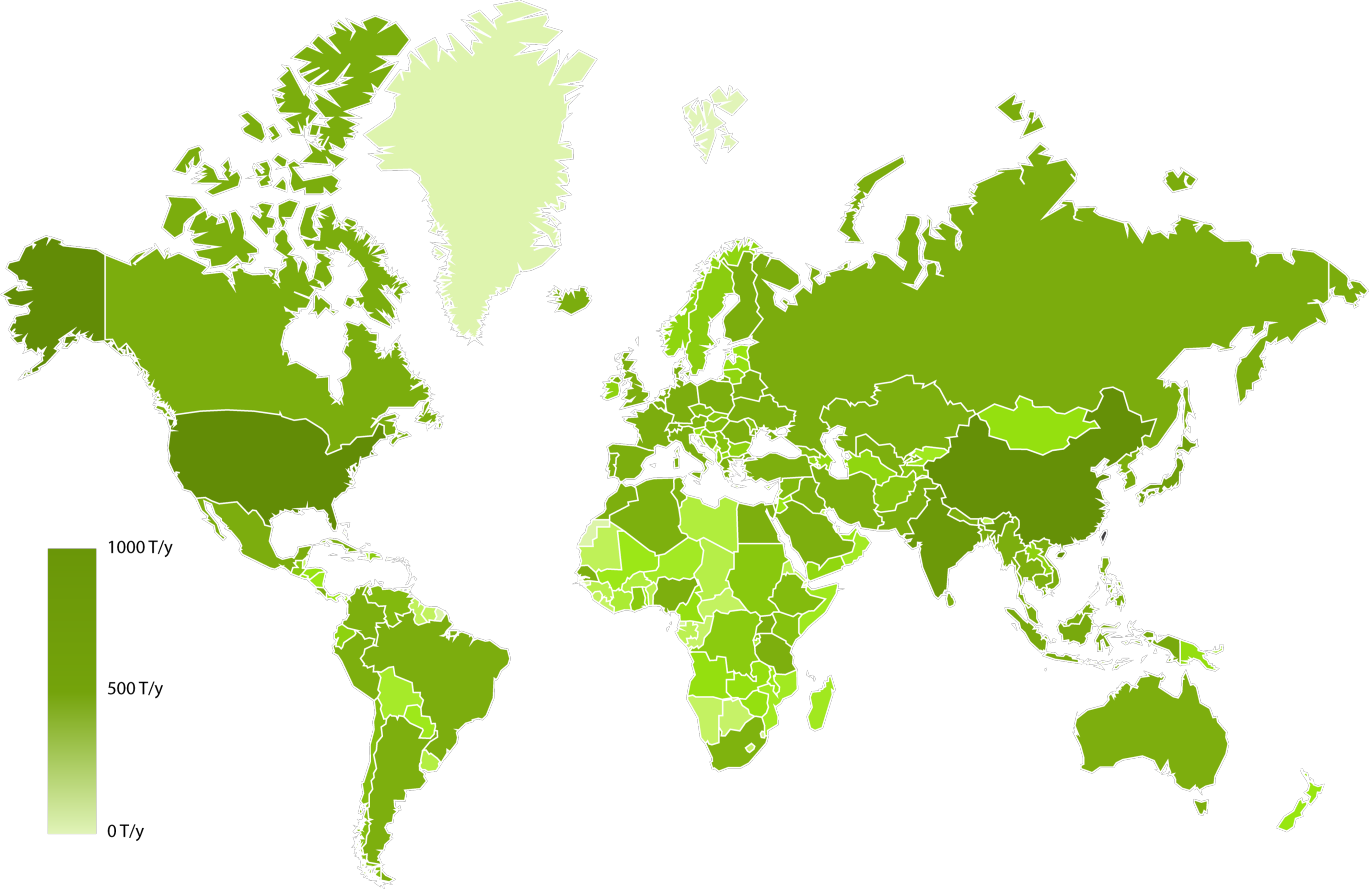
Example of the global distribution of the release rates of TiO_2_ into water.

Implementation of the LearNano model includes user guidance and visualization tools for data input and simulation results, a model solver, and a parameter database. The analysis scenario (i.e., a given combination of ENM, region, and application(s)) is constructed within the GUI, which also captures ENM production rates and the various transfer coefficients between adjoining compartments (both technical and environmental). ENM production rates and transfer coefficients can be obtained from a parameter database by specifying the ENM(s), application(s), and region(s) of interest (see section, Databases). The mass balance equations (Supporting Information File 1, Equations S1–S4) are then solved to determine the average ENM release rates to the environmental compartments (i.e., air, water, and soil). Mass “flows” of ENMs among the various compartments can be visualized using a dynamic and interactive Sankey diagram (Figure 4). Also, the global distribution of ENM release (to various environmental compartments) in different countries can be represented on a world map (Figure 5). It is noted that, while the present version of LearNano computes ENM release rates on a country level, estimates of regional ENM release rates may be obtained by scaling country level release rates on the basis of population, area, or economic indicators [7,17].

### Graphical user interface (GUI)

**Figure 6:**
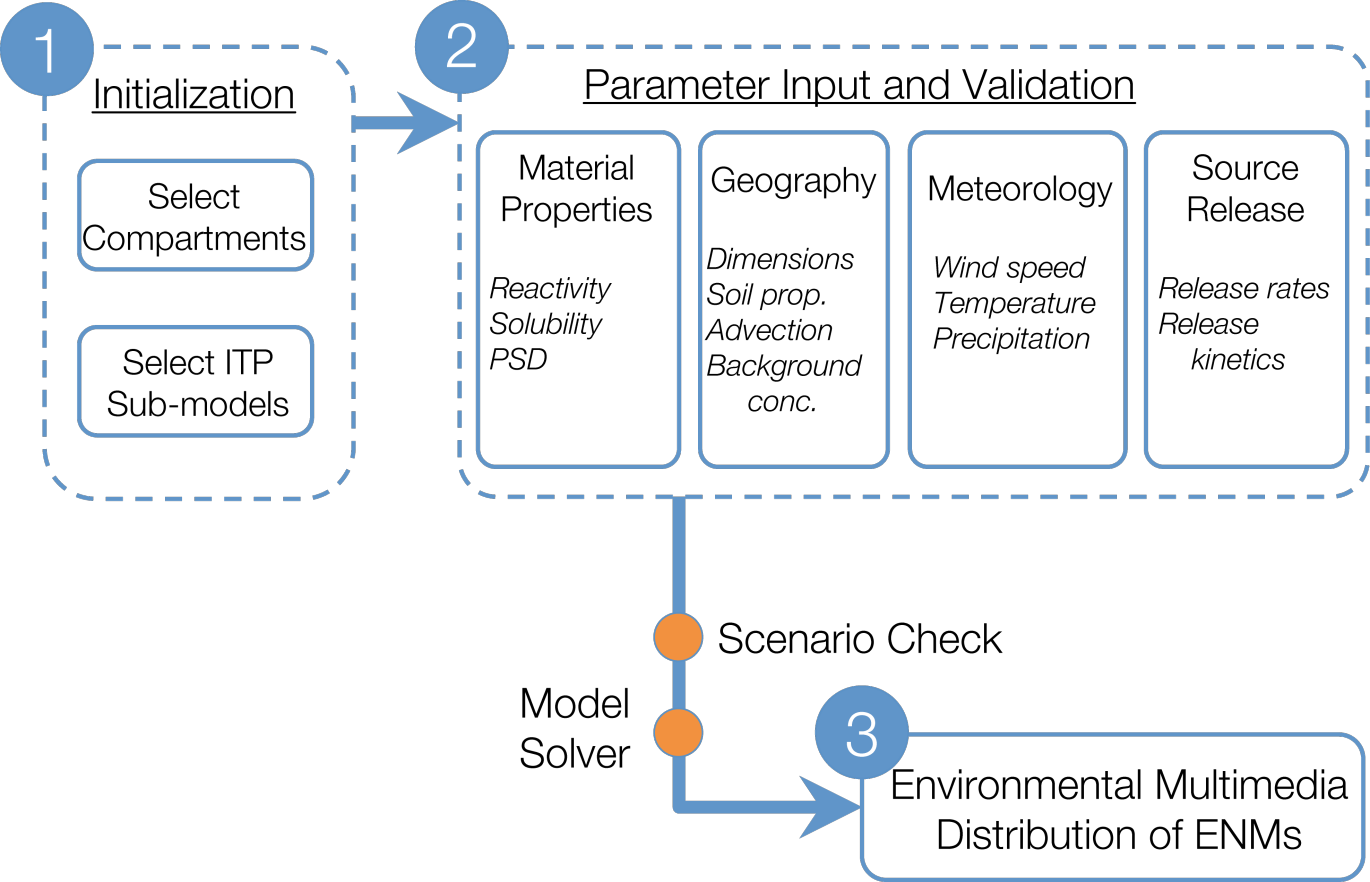
Workflow for assessing the environmental distribution of ENMs. ITP: intermedia transport processes, PSD: particle size distribution.

The web-based GUI for RedNano enables building multimedia scenarios, initiating model execution, as well as visualization of simulation results. A multimedia scenario refers to the specification of a model environment (i.e., geographical region and its meteorology), the target ENM, and its release rate. A multimedia scenario is built by specifying or selecting the required parameters from modules that include: (a) geography, (b) meteorology, (c) material properties, and (d) source release (Figure 6).

Scenario design is initiated by selecting the environmental compartments (e.g., air, water, soil, sediment, vegetation canopy, biota) and ITPs (e.g., dry/wet deposition, resuspension, sedimentation, dissolution) of interest for the desired simulation period (typically ≈1 year) and the target ENM and its properties (Figure 6). Subsequently, submodels are selected for the specified ITPs (Figure 2) and the regional geographical and meteorological parameters are specified for the selected region (Figure 6). The values for these parameters may be obtained from the system’s parameter database, or can be provided by the user. ENM release rates to the various compartments are also required and these can be obtained from LearNano by selecting the target ENM, region, and applications of interest, or specified directly by the user (Figure 6). The temporal profile of the ENM release rate kinetics can be specified as constant or periodic sinusoidal (e.g., to mimic seasonal and diurnal variability).

**Figure 7:**
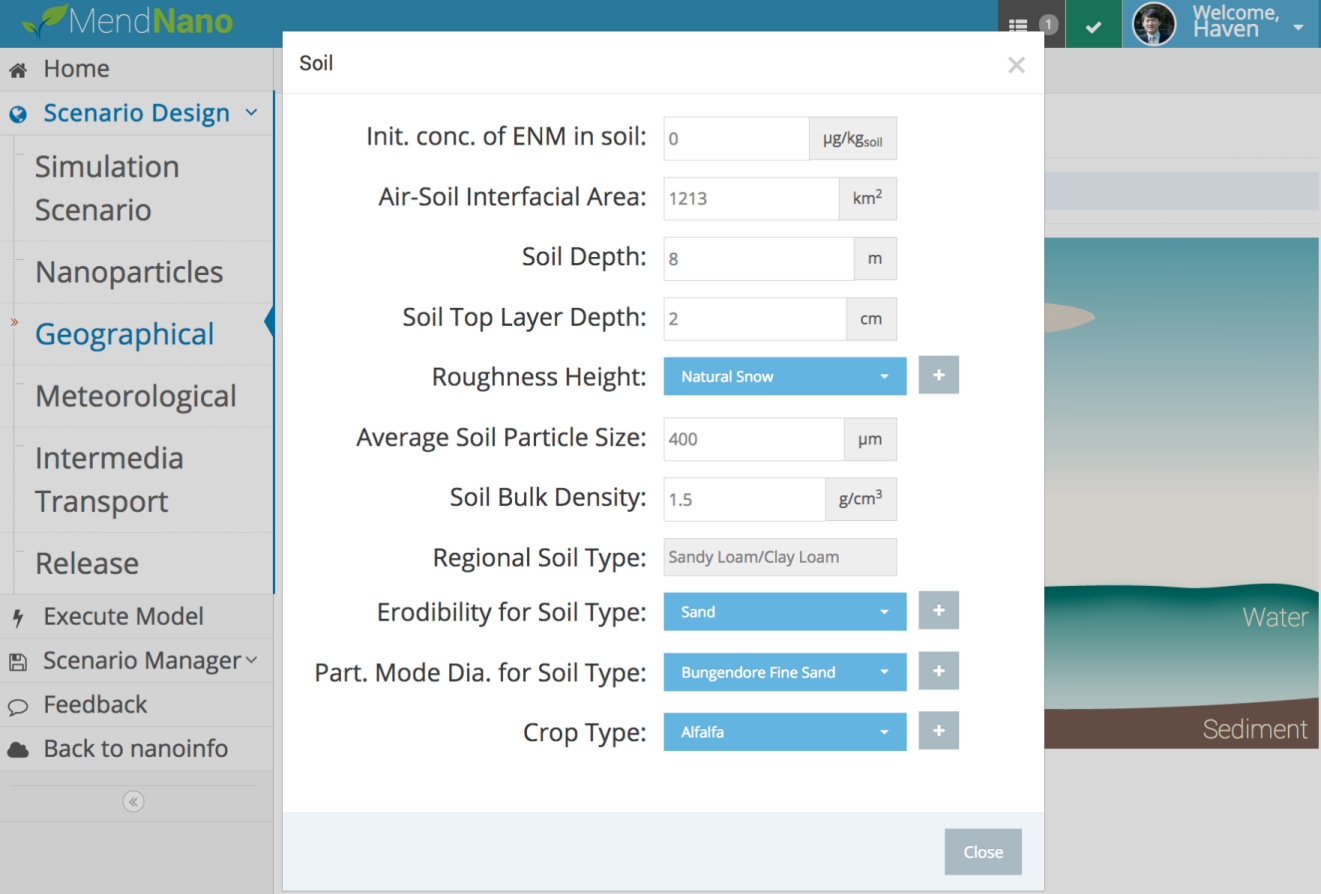
Examples of MendNano web-based graphical user interface for scenario building showing inputs of soil parameters.

**Figure 8:**
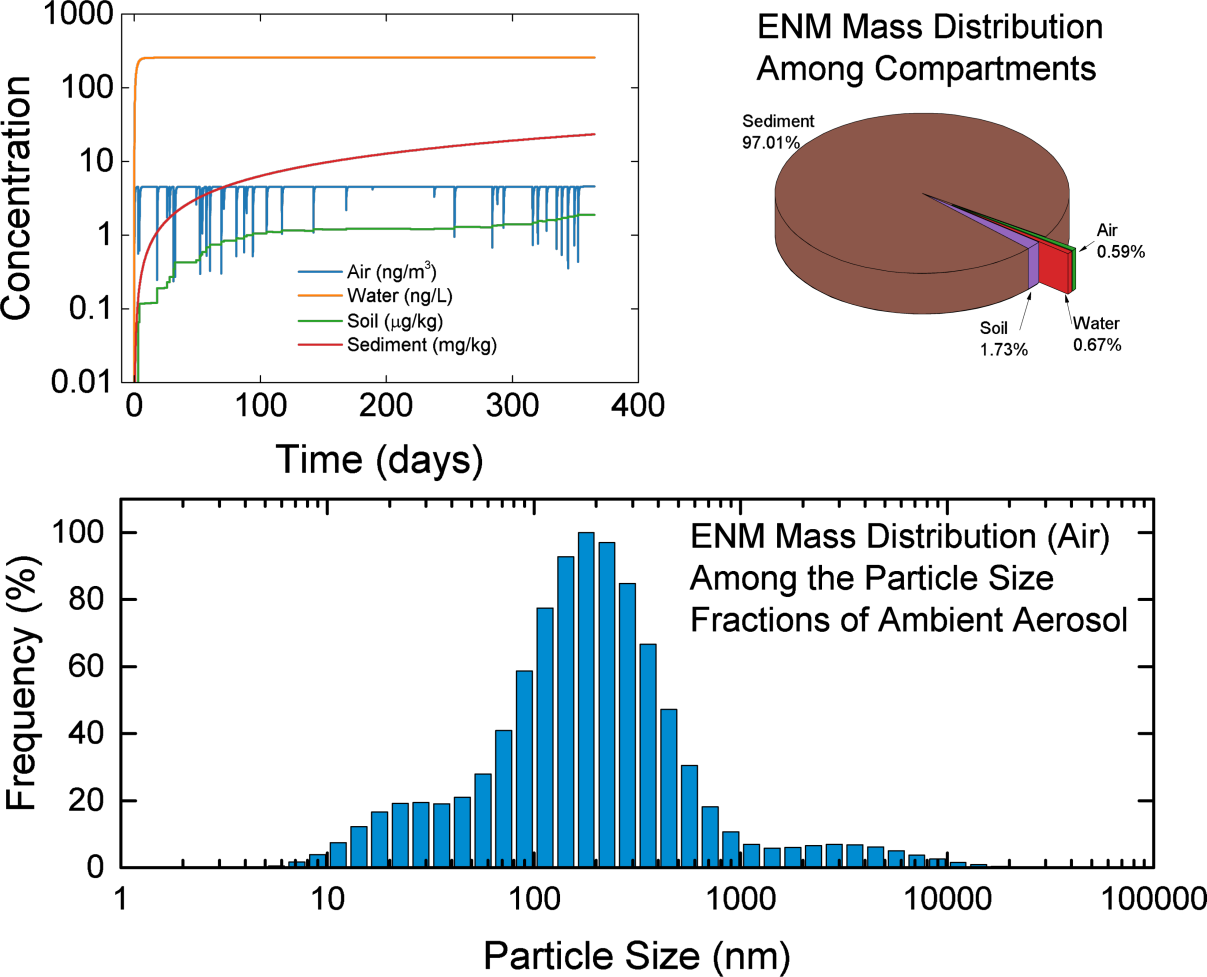
Examples of graphical representations of MendNano simulation results depicting concentration profiles and mass distributions of TiO_2_ in the Los Angeles region among the various compartments and among the ambient particles in air. Release of TiO_2_ in the above example is in air (5,000 kg yr^−1^) and water (19,381 kg yr^−1^).

The specification of the required parameter values is accomplished in a series of web pages (or views; Figure 7) within the GUI corresponding to the modules shown in (Figure 6). The parameter input is validated, prior to model execution, to ensure that the specified values are within a reasonable range and/or constraints (e.g., minimum regional area, maximum rainfall intensity). Additional simulation scenario validation is also conducted to ensure that scenarios are not ill-defined (e.g., simulation with neither source release nor initial compartmental concentration). Upon simulation scenario design completion, model execution is initiated (a unique Simulation ID is assigned for compilation of a scenario library). The results can then be visualized via a series of graphical representations. The dynamic multimedia ENM distributions can be represented as: (a) ENM temporal concentration (or mass) profiles in various compartments (Figure 8), (b) intermedia mass transport rates or fluxes, (c) ENM mass distribution (percent) among the various compartments, (d) ENM apportionment throughout the ambient particle size distribution (Figure 8), and (e) the magnitude of intermedia transport rates, as a fraction of the ENM release rates, that allows assessment of the relative significance of various intermedia transport processes (Supporting Information File 1, Figure S5). For example, in the illustration of Figure 8, ENM concentrations in air and water (left upper plot) rapidly reach pseudo-steady state, except during episodic rain events, in which a sharp decrease in ENM concentration in air is observed, followed by a rapid increase after the rain event. In contrast, ENM concentrations in soil and sediment continue to increase, since ENM removal rates from soil and sediment are significantly lower than the rate of ENM entering the soil and sediment. Given these considerations and that the ENM release rate to water was greater relative to air (Supporting Information File 1, Figure S5, Table S5), the majority of ENM mass accumulated in the sediment (right upper subplot). The ENM mass distribution in air among the particle size fractions of ambient aerosol is shown to follow the expected tri-modal distribution (lower subplot). It is noted that such information can be utilized to convert MendNano reported ENM mass concentrations to surface area concentration [35–36] given the knowledge of the primary particle size.

### Databases

The parameter database contains material properties, geographical, and meteorological parameter values (Table 1), which are compiled from various literature and database sources [31,37–39]. The parameter database also includes a library of ENM production rates and transfer coefficients corresponding to specific ENMs and applications, for different geographic regions (Table 1), compiled from various published studies [17], public databases [40], and market research [5], and estimated based on economic indicators [41]).

**Table 1:** Parameters database.

Category	Subcategory	Property^a^

Material properties		PSD (ENM and aerosol)
Geographical parameters	Physical description	Interfacial Area (air–water, air–soil)
		Mixing height
		Water depth
		Water flow rate
		Average suspend solids diameter
		Sediment depth
		Soil depth
	Dry deposition to vegetation	Roughness factor
		Characteristic field length
		Crop vegetation factor
	Dry deposition to soil	Roughness height
	Wind resuspension of soil	Soil erodibility
Meteorological parameters		Monthly Temperature (air, water)
		Wind speed (monthly, annual average, max)
		Rainfall rate (monthly)
LearNano parameters		ENM Global production rate
		Transfer coefficients (ENM specific)
		Transfer coefficients (application specific)
		Transfer coefficients (region specific)

^a^Additional parameters, including those calculated internally by the model, are provided in Supporting Information File 1, Table S1.

### Use cases for assessing multimedia distribution of ENMs

The integrated RedNano simulation tool is suitable for a variety of assessments regarding the environmental distribution of ENMs and their fate and transport behavior. These assessments can be classified into use cases that include, but are not limited to, the following:

1. Environmental ENM concentrations and mass distribution based on a specified multimedia scenario;
2. Dynamic response of the environmental system to temporally varying ENM release rates;
3. Impact of specific intermedia transport processes on the temporal dynamics of ENM distribution in the environment;
4. Comparison of estimated environmental ENM concentrations in various regions;
5. Contribution by ENM applications (or use) to the overall ENM releases and exposure concentrations in the various environmental compartments;
6. Estimation of source release rates, based on matching of model estimates and reported environmental concentrations.

## Results and Discussion

**Figure 9:**
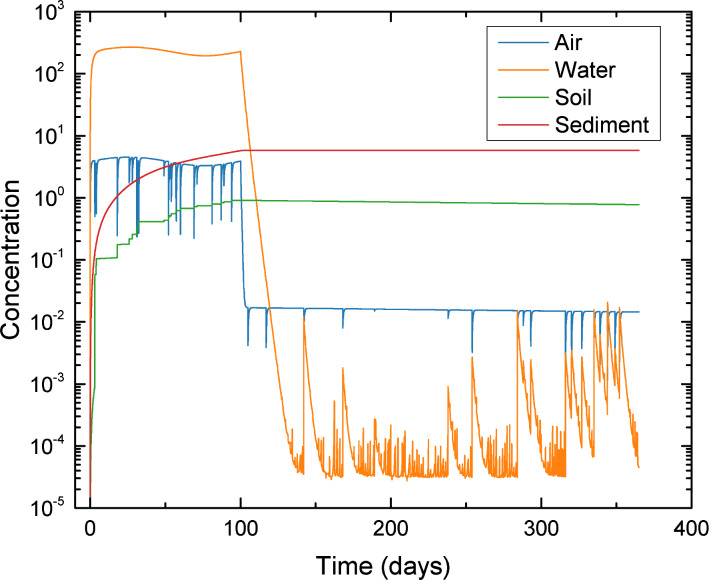
Effect of release scenario on temporal dynamics of TiO_2_ media concentrations in Los Angeles. TiO_2_ release rates to air and water were obtained from LearNano (Supporting Information File 1, Table S5). The ENM release rates (into air and water) followed a sinusoidal function for the first 100 days (cycle period of 100 days, amplitude of 13.7 and 53.1 kg/day, for releases to air and water, respectively), after which the source releases are terminated. Regional geographical parameters are reported in Supporting Information File 1, Table S4.

In order to demonstrate the above use cases, illustrative simulations were conducted to estimate the environmental distributions of TiO_2_, CeO_2_, SiO_2_, and CNT in selected regions. The multimedia distribution of ENMs (use case #1) and the dynamic response of an environmental system to temporal variations of ENM release rate (use case #2) are illustrated for TiO_2_ in Los Angeles. Due to a lack of transfer coefficients specific to Los Angeles, TiO_2_ release rates for Los Angeles were estimated by scaling from US release rates on the basis of a population ratio. TiO_2_ release rates to air and water were taken to follow a sinusoidal release function with a cycle period of 100 days, where the release rates fluctuated between 0 to 27.4 and 0 to 106.2 kg day^−1^, for release into air and water, respectively, and were terminated thereafter. The results, as shown in Figure 9, indicate that TiO_2_ concentrations in air and water fluctuate between 3.3–4.4 ng m^−3^ and 195–267 ng L^−1^, respectively, representing an ≈15% deviation (in both media) above and below the time-averaged concentration in the respective compartments. Following cessation of source release into air and water (at *t* = 100 days), the TiO_2_ concentration in both compartments decreased rapidly (Figure 9) to 90% of the levels just prior to the termination of the release in ≈1 day and ≈4 days, respectively. The TiO_2_ concentrations continued to decrease until a pseudo-steady state was reached in air and water, within ≈4 and ≈38 days, respectively. Although ENM release into air and water ceased after 100 days, the ENM concentrations in these compartments did not vanish since ENMs in the soil (accumulated during the first 100 days) continued to be transported to air via soil–wind resuspension, and subsequently deposited to water via dry and wet deposition.

The impact of specific intermedia transport processes on the temporal dynamics of the ENM distribution in the environment (use case #3) is highlighted via a series of simulations for TiO_2_ in Los Angeles focusing on intermedia transport via dry deposition, rain scavenging, and wind dilution (Supporting Information File 1, Figure S1). In these scenarios, the initial TiO_2_ concentration in air is taken to be the steady state TiO_2_ concentration reached after 1 year with all other compartments being initially free of TiO_2_.

**Figure 10:**
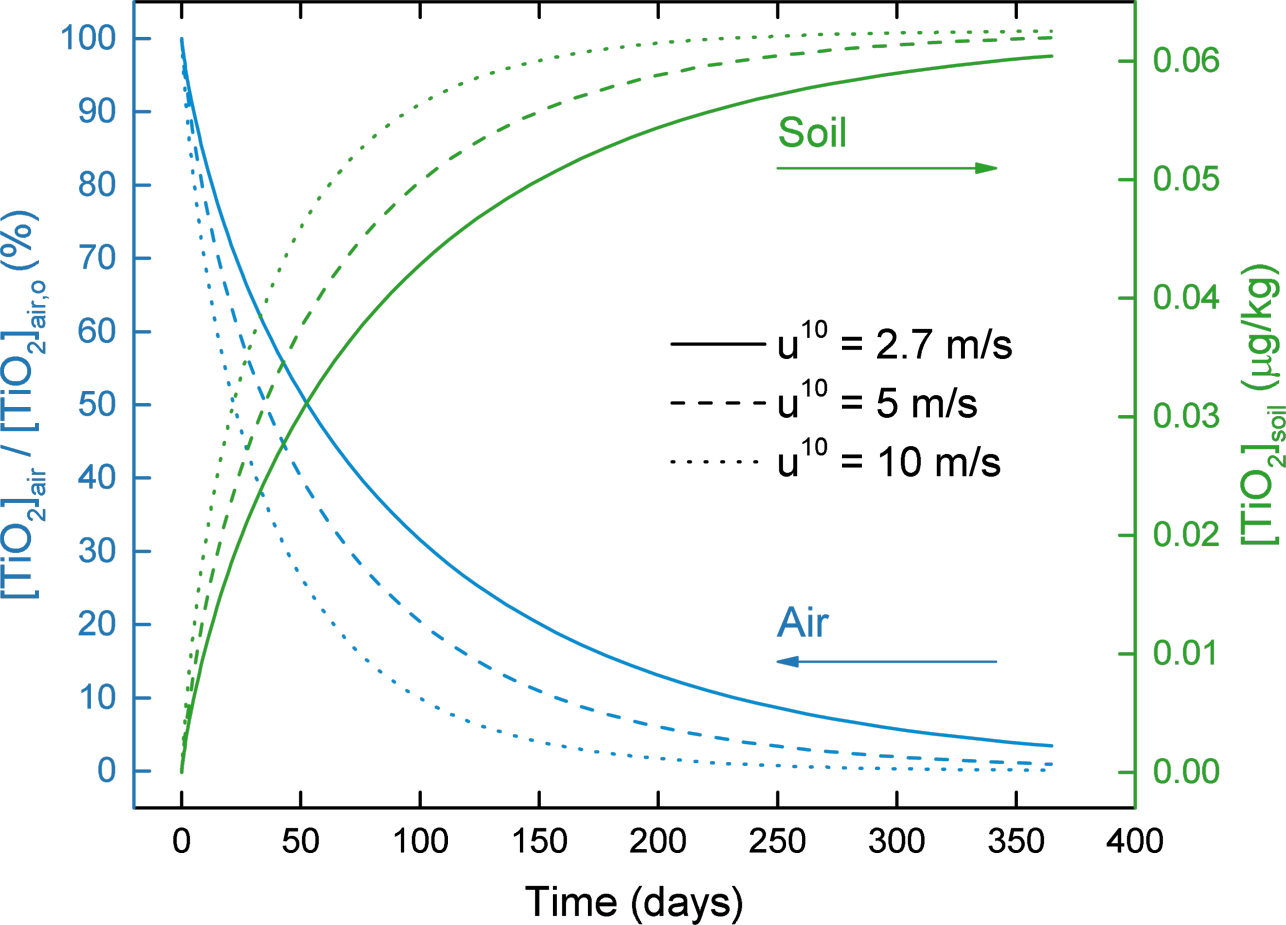
Effect of dry deposition on the reduction of TiO_2_ concentrations in air and soil (postcessation of all ENM releases) in Los Angeles as a function of wind speed (range of 2.7–10 m s^−1^). Regional geographical parameters are reported in Supporting Information File 1, Table S4.

Dry deposition is a process in which particles (including ENMs) are collected onto terrestrial (e.g., soil, vegetative canopy) and aquatic surfaces due to Brownian diffusion, impaction, and interception [42]. The intermedia transport rate due to dry deposition is a function of wind speed (among other parameters, e.g., surface roughness), which is typically reported to be 3.3 ± 0.95 m s^−1^ (1 standard deviation for 1996–2006) [43], with a maximum of ≈10 m s^−1^ in the Los Angeles region (LAX station). An increase in wind speed would lead to an increase in the rates of collection by impaction and interception [42], and thus an increase in the overall rate of dry deposition. The predicted temporal ENM concentration profiles in air and soil (Figure 10) reveal that the time to remove 90% of TiO_2_ by dry deposition alone is ≈100–230 days for wind speed in the range of 2.7–10 m s^−1^. Additionally, at the end of a 1 year simulation, 0.1–3.4% of the initial ENM mass in air remains in the air compartment for this wind speed range.

**Figure 11:**
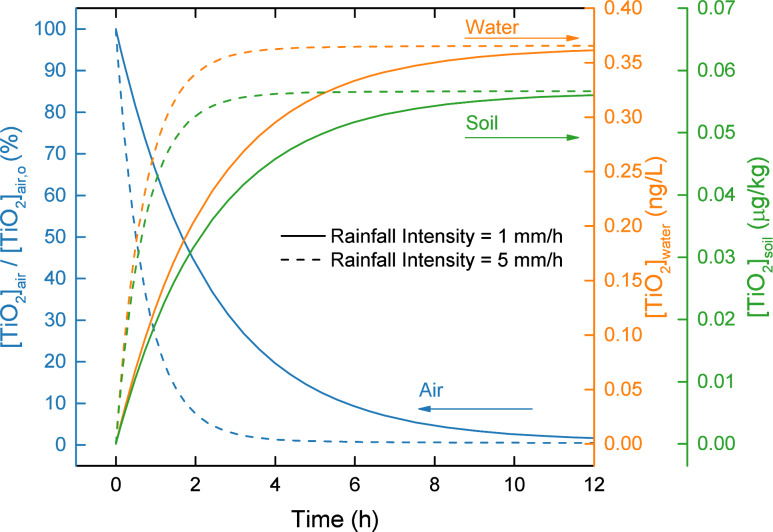
Effect of rain scavenging on TiO_2_ concentration in air, water, and soil in Los Angeles as a function of rainfall intensity (1–5 mm h^−1^). All ENM release rates are terminated at the start of a long rain event, which was taken to last for 12 h. Regional geographical parameters are reported in Supporting Information File 1, Table S4.

Rain scavenging of particulate matter (including ENMs) by raindrops results in the removal of particulate matter from the atmosphere and its subsequent deposition onto terrestrial and aquatic surfaces. The ENM removal rate by rain scavenging is governed by rainfall intensity (typically in the range of 1–10 mm h^−1^ for light to moderate rain [44], and can exceed 50 mm h^−1^ for intense storms [45]). Rain scavenging can typically remove atmospheric particles at a faster rate relative to dry deposition. As illustrated in Figure 11, even with a mild rainfall intensity of 1–5 mm h^−1^, 90% of TiO_2_ can be removed in hours (i.e., ≈2–6 h, corresponding to a rainfall intensity of 5–1 mm h^−1^), compared to many days for removal by dry deposition (Figure 10). Since rain scavenging is an episodic process (in contrast to the continuous dry deposition), the annually averaged ENM removal rate by rain scavenging is expected to be lower than the instantaneous removal rate during rainfall events as shown in Figure 11. Nonetheless, the averaged transport rate by rain scavenging can exceed that by dry deposition. For example, in Los Angeles, the estimated annually averaged TiO_2_ removal by rain scavenging is a factor of ≈10 greater than by dry deposition (Supporting Information File 1, Figure S5), indicating that rain scavenging has a more significant impact on the environmental ENM distribution relative to dry deposition.

**Figure 12:**
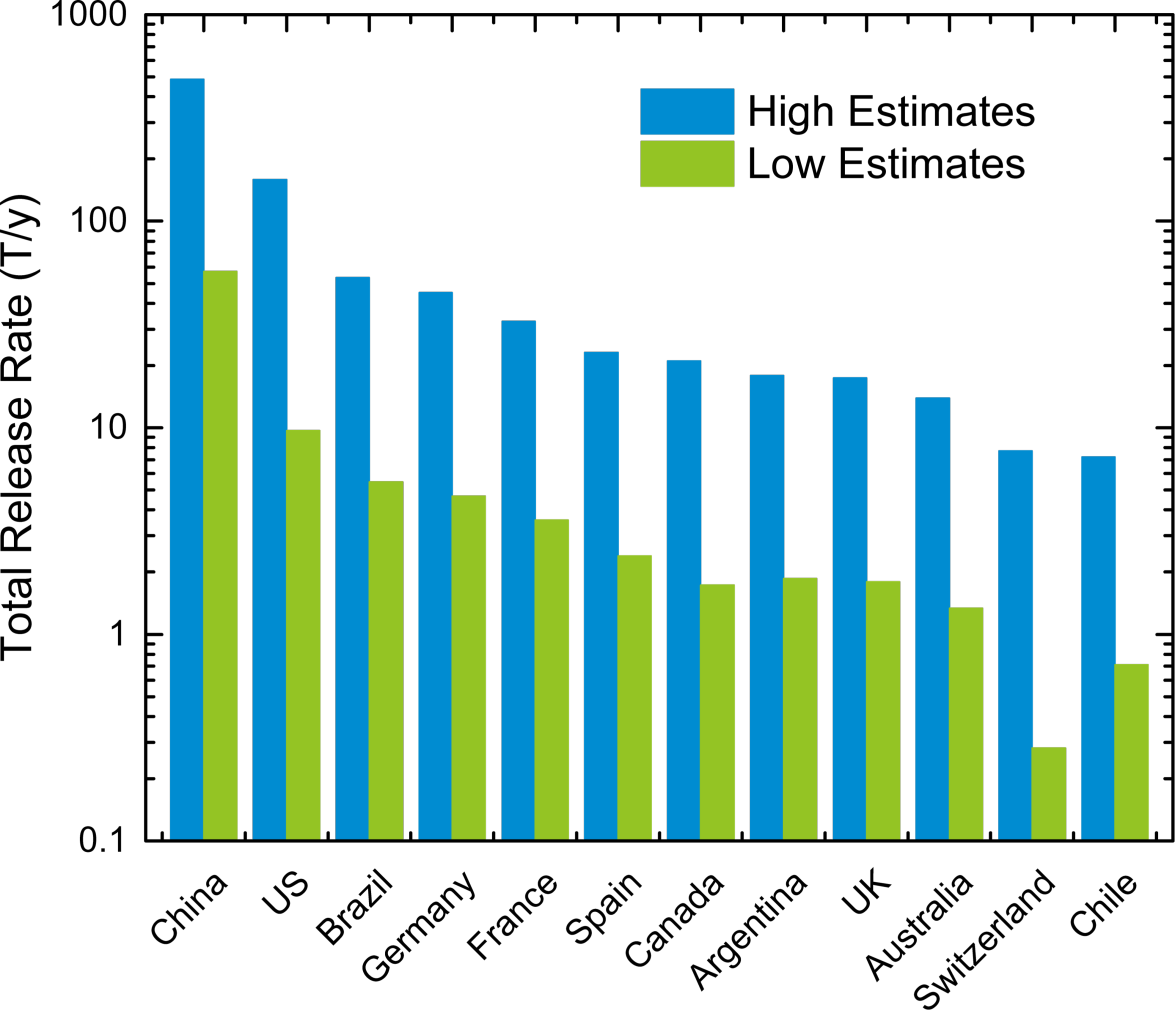
Estimated CeO_2_ release rates for 12 selected countries.

A comparative analysis of the potential environmental ENM concentrations in various countries (use case #4) is given using the example of CeO_2_ ENMs, whereby release rates were estimated via LearNano for 12 selected countries. These countries were selected to represent the high ENM producing (and high emission) regions. The estimated CeO_2_ release rates (high estimate) for the 12 countries span over the range of 7.2–486 T yr^−1^ for Chile and China (Figure 12). The high estimates for the release rates for the 12 countries are, on average, a factor of ≈12 greater than the low estimates, with the highest difference being by a factor of 86 (e.g., for release to water in Switzerland). The release rates into air, water, and soil represent, on average for the different countries, 10% (3–40%), 38% (33–46%), and 52% (24–60%) of the total release rates, respectively (Supporting Information File 1, Figure S2). The above analysis suggests that while some differences exist in apportionment of total release to various compartments between countries, the majority of ENM release events are into water, followed by soil and air. It should be noted that among the total ENM release to soil, only the direct release portion (≈79%, which excludes release from WWTP biosolids) may be considered to be distributed over the entire soil area in the region. The distinction between direct release to soil and that from WWTP biosolids is important. Although biosolids are applied to some agricultural lands in the USA, the USEPA estimates that <1% of agricultural lands receive biosolids [46], which suggests that the application of biosolids to soil does not represent a wide spread release in the USA. Similarly, it has been reported that in Switzerland, biosolids are not applied to soil, and are instead processed in waste incineration plants [17].

**Figure 13:**
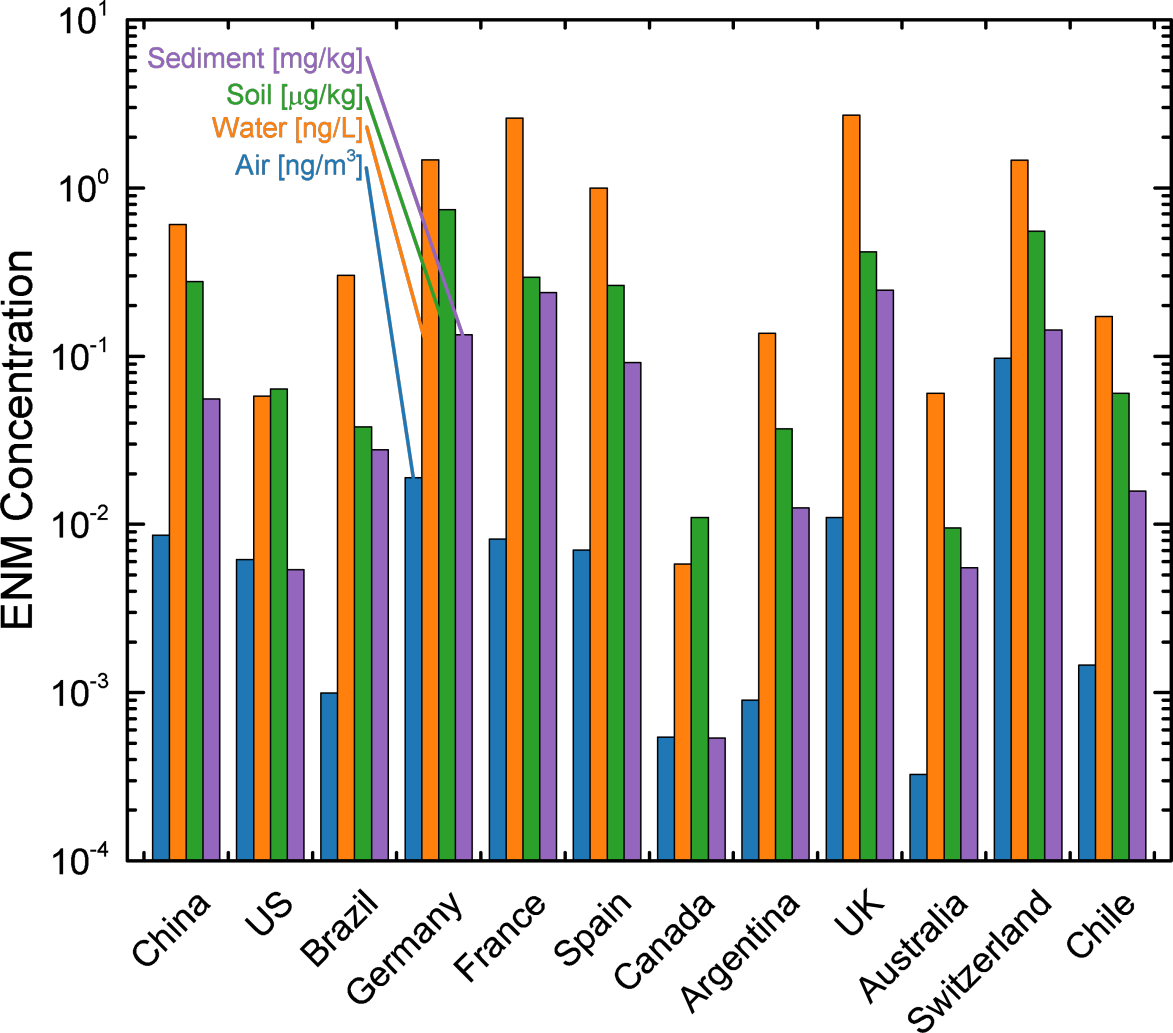
Predicted compartmental concentrations for CeO_2_ in 12 selected countries at the end of a 1 year simulation for the ENM release rates reported in Figure 12**.** Regional geographical and meteorological parameters are reported in Supporting Information File 1, Table S4.

The compartmental concentrations of CeO_2_ for the 12 countries were estimated via MendNano using the release rate estimates shown in Figure 12, and country specific geographical and meteorological conditions (Supporting Information File 1, Table S3). The simulations were carried out assuming that only direct release to soil is regionally distributed. The predicted CeO_2_ concentrations using the high release rates estimates are in the range of 0.0003–0.097 ng m^−3^, 0.0058–2.7 ng L^−1^, 0.0095–0.74 μg kg^−1^, and 0.0054–0.25 mg kg^−1^ for air, water, soil, and sediment, respectively (Figure 13). Relative to these predictions, the CeO_2_ concentrations predicted using the low release rates estimates are a factor of 5–1243 lower (Supporting Information File 1, Figure S3). Clearly, there is a large uncertainty in the estimated media concentrations due to uncertainties in ENM release estimates. Nonetheless, it is noted that the above predicted CeO_2_ concentration range is significantly below concentrations typically used in experimental toxicity studies [47].

It is interesting to note that while the USA ranks second highest in terms of release rates (for all compartments), it ranks 7th (out of 12) in terms of CeO_2_ concentration in air and soil, and 11th based on concentration in water and sediment. In contrast, while the UK and Switzerland rank 9th and 11th with respect to total release rates, respectively, they rank first (i.e., highest) in terms of the compartmental concentrations in air and water, respectively. Additionally, the environmental concentrations in the European countries are all significantly higher than that in the US (by a factor of 1.4–15), despite having total release rates that are lower than the USA (by a factor of 3.5–20). The apparent resulting discrepancy between release and environmental concentrations is attributed to differences in geography and meteorology. For example, Supporting Information File 1, Figure S4 shows that the release rate into air per unit area (combined soil and water) in Switzerland is a factor of 17 greater than in the US; similarly, release rates into water per unit area in the UK are a factor of 46 greater than in the US.

**Figure 14:**
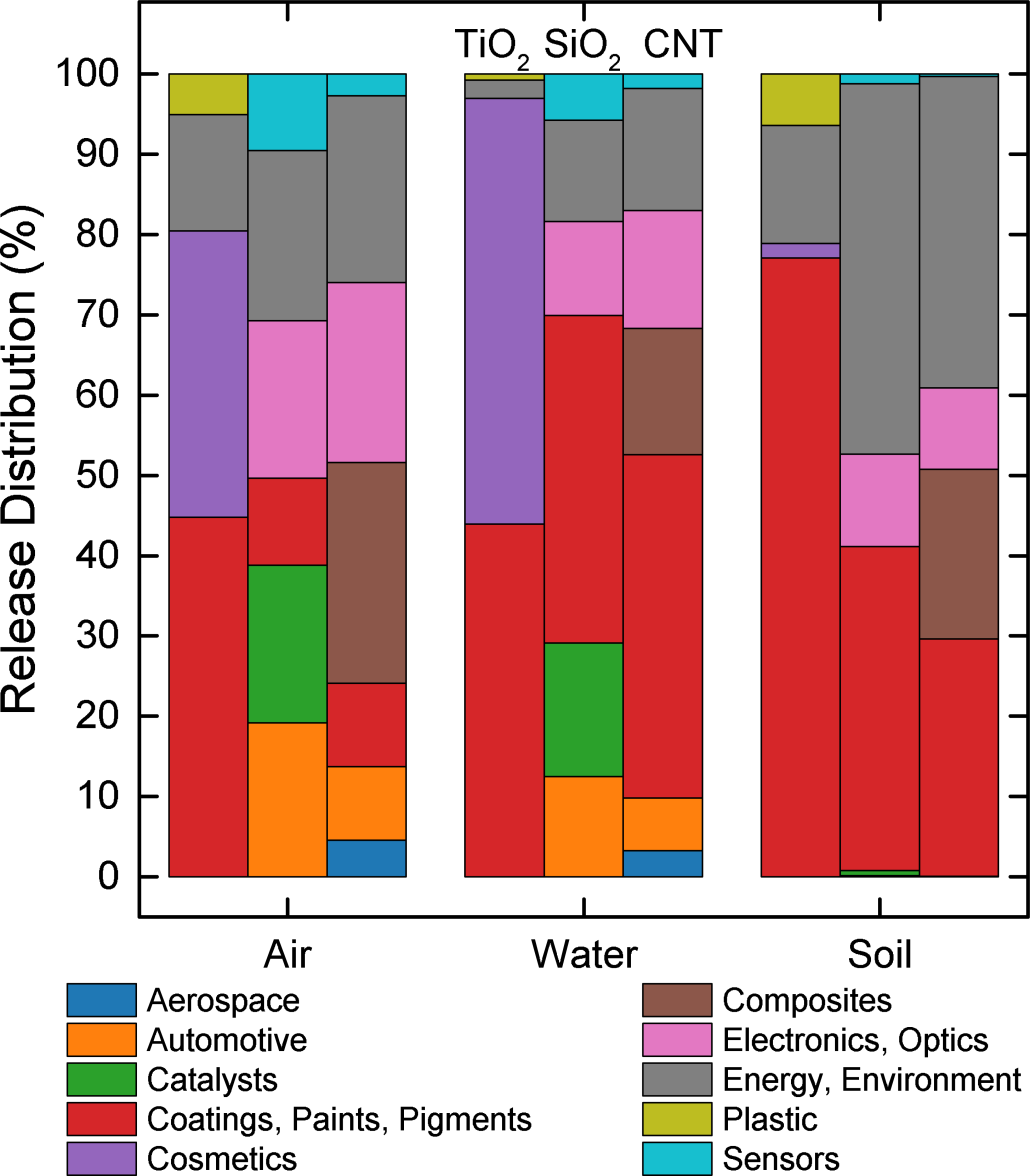
Apportionment of environmental release rates of selected ENMs to specific compartments in the Los Angeles region to different ENM applications.

The contribution of ENM release rates by various ENM applications (or use) to the overall ENM release and exposure concentrations in the various environmental compartments (use case #5) is shown in the example of Figure 14 and Supporting Information File 1, Figure S6. For Los Angeles, the simulations were carried out for TiO_2_ and SiO_2_, which were selected since these are produced in the largest quantity [7], and CNT was included due to its diverse applications [7]. The TiO_2_ release rates attributed to coating, paint, and pigment applications are the primary contributors of the release of this ENM into air (≈45%) and soil (≈77%). In water, TiO_2_ release is associated with cosmetic applications, which represent the largest fraction at ≈53%, while those associated with coatings, paints, pigments represent ≈44%, with remainder due to energy applications (e.g., photovoltaics, energy storage [7]), environmental (e.g., remediation [7]), and plastic applications. These results are consistent with reported TiO_2_ use in coatings, paints, and pigments and associated release into the environment due to weathering [48] and TiO_2_ used in cosmetics is primarily released during washing into waste water [49]. The release of SiO_2_ into air (Figure 14) associated with energy and environmental applications is the largest fraction (≈21%), while other applications (i.e., automotive, catalysis, coatings/paints/pigments, electronics/optics, and sensors) contribute less, but still a significant amount (9.5–19.6%). In contrast, the release of SiO_2_ into soil is dominated by energy and environmental applications, and the group of coating, as well as paint and pigment applications (46% and 40%, respectively), while other applications collectively contribute less than 14% of the total SiO_2_ release to soil. The most significant contribution to SiO_2_ released into water is also associated with coating, paint, and pigment applications (≈41%). Finally, the largest contributions to the release of CNTs into air, water and soil are associated with composites (≈28%), coatings, paints and pigments (≈43%), and energy and environmental applications (≈40%), respectively.

The contributions of the various ENM applications to compartmental concentrations (Supporting Information File 1, Figure S6) are, as expected, typically qualitatively similar to their contributions to the ENM release rates shown in Figure 14. However, noticeable differences can be observed in some cases due to intermedia transport of these ENMs from soil to air. For example, an ENM associated with a given ENM application can be transported to the air compartment via soil–wind resuspension in larger portion relative to other applications. Thus, increased ENM concentration in air may occur for that application. Such a behavior can be expected when an ENM application contributes to the ENM release to soil in larger proportion relative to its contribution to ENM release to air. The above behavior is demonstrated in Supporting Information File 1, Figure S6 for TiO_2_, for which the release associated with coatings, paints and pigments contributes ≈45% to the total TiO_2_ release to air while contributing ≈77% of total TiO_2_ release to soil (Figure 14). As a result, ≈54% of the TiO_2_ mass concentration in air is attributed to releases associated with coatings, paints, and pigments. In contrast, when 36% of the total TiO_2_ release to air is associated with cosmetics applications, and only 1.8% of total TiO_2_ release to soil is associated with cosmetics, less than 28% of the TiO_2_ mass concentration in air is related to this category of ENM application. Therefore, since wind resuspension from soil may be a significant transport pathway of ENMs into the air compartment, the apportionment of the total ENM release to soil associated with the various applications may have a notable impact on the contribution of ENM application to its concentrations in air.

The estimation of ENM release rates, based on reported environmental ENM concentrations (use case #6), can be accomplished as described in the example of simulations of CeO_2_ environmental distribution in Newcastle (UK). In this example, the release rate of CeO_2_ ENMs from fuel additives in Newcastle was estimated based on matching reported atmospheric concentrations before and after the introduction of the fuel additive with MendNano simulation results. Monitoring the results showed that following the introduction of Envirox (a CeO_2_ ENM-based diesel fuel combustion catalyst) to a bus fleet in the Newcastle area, the ambient CeO_2_ concentration increased by a factor of ≈4.2 (0.574 ng m^−3^, from 0.145 to 0.612 ng m^−3^) [50]. MendNano simulations carried out considering the geographical and meteorological scenario setup for the Newcastle region revealed that a CeO_2_ release rate of 43.96 kg yr^−1^ would result in the reported increased CeO_2_ concentration. The MendNano estimate of the CeO_2_ release rate is consistent with the release rates estimated based on: (a) vehicle miles travelled (VMT) and (b) the diesel fuel consumption rate in the region of Northumberland, which is in proximity to Newcastle and of similar population (Supporting Information File 1, Estimation of CeO_2_ Release Rates in Newcastle UK by VMT and Diesel Fuel Consumption). The estimated CeO_2_ release rates for the above two cases are 21.48 and 44.82 kg yr^−1^, respectively.

## Applications and Merits

In summary, an integrated release and environmental distribution of nanomaterial (RedNano) simulation tool was developed and implemented as a web-based application to enable rapid “what-if?” scenario analysis. The RedNano simulation tool is suitable for both research as well as educational purposes, and can be utilized in both undergraduate and graduate level courses for multimedia environmental assessment. It is envisioned that the present multimedia analysis platform can assist regulators, industry, and researchers to rapidly assess the potential environmental implications of ENMs that may be released into the environment.

## Supporting Information

File 1 Additional equations and results regarding the model equations, intermedia transport factors, use cases, and parameters used for simulations carried out in the study.
