# Supplementary material for: Simulation tool for assessing the release and environmental distribution of nanomaterials
Source: Beilstein J Nanotechnol. 2015 Apr 13;6:938–51. doi: 10.3762/bjnano.6.97 (PMC4419581; doi:10.3762/bjnano.6.97)
Supplement: File 1 — Additional equations and results regarding the model equations, intermedia transport factors, use cases, and parameters used for simulations carried out in the study. [file Beilstein_J_Nanotechnol-06-938-s001.pdf]

Supporting Information

for

Simulation tool for assessing the release and environmental distribution of nanomaterials

Haoyang Haven Liu[1,2*], Muhammad Bilal[1], Anastasiya Lazareva[3], Arturo Keller[1,3], and Yoram Cohen[1,2*]

[1]Center for the Environmental Implications of Nanotechnology, California NanoSystems Institute, University of California, Los Angeles, CA 90095, USA, [2]Chemical and Biomolecular Engineering Department, University of California, Los Angeles, Los Angeles, CA 90095, USA, and [3]Bren School of Environmental Science & Management, University of California, Santa Barbara, Santa Barbara, CA 91306, USA

Email: Yoram Cohen* - yoram@ucla.edu; Haoyang Haven Liu* - haven.liu@ucla.edu

*Corresponding author

Additional Equations and Results

Introduction

This supporting information provides additional details regarding the multimedia mass balance equations, ENM lifecycle mass release equations, the use cases described in the main text, estimation of CeO_2 ENM release rates from diesel fuel additive, and assessment of the effect of wind dilution on dynamics of TiO_2 concentration in air in Los Angeles. Also, tables are provided with additional intermedia transport factors, parameters used for simulations carried out in the study, and estimated release rates of TiO_2, SiO_2, and CNT in Los Angeles. Lastly, additional results are provided to illustrate the use cases discussed in the main text.

Multimedia Mass Balance Equations

The mass balance equations, which incorporate intermedia transport rates, serve to compute the ENMs concentration (and mass) in various environmental compartments as a function of time, and for the complete particle size distribution discretized as N size fractions:

$$\frac{d}{dt}m_{i,k} = \frac{d}{dt}\left[V_i C_{i,k}\right] = \left(Q_i^{in} C_{i,k}^{in} - Q_i^{out} C_{i,k}\right) + \sum_{\substack{j=1 \\ j \neq i}}^{M} \sum_{l=1}^{P} I_{i,j,k}^{l} + \sum_{n=1}^{U} R_{i,k}^{n} + S_{i,k} \qquad \begin{matrix} k=1\ldots N; \\ i=1\ldots T \end{matrix} \qquad [1]$$

where $m_{i,k}$ is the mass (kg) of the ENM of size fraction k in compartment i, V_i is the volume (m^3) of compartment i, and $C_{i,k}$ (g m^{-3}) is the concentration of ENM of size fraction k in compartment i. Typically, $N = 50$ size fractions are used to discretize each of the PSDs of ENMs and ambient particles in air and water. The first term on the right hand side (RHS) of Equation 1 is the advective flow transport, where Q_i is the convective flow rate in (with superscript *in*) or out (with superscript *out*) of compartment i. The second term on the RHS describes the ENM intermedia transport between compartments i and j (Figure 2), where intermedia transport rate between compartment i and j, via transport process l, given by $I_{i,j,k}^{l}$ (g s^{-1}), is summed over all processes (P) from all compartments (M). The third term on the RHS represents various reaction (and dissolution), where $R_{i,k}^{n}$ (g s^{-1}) is the transport rate, and the $S_{i,k}$ (g s^{-1}) is the source release rate.

Table S1: Basic intermedia transfer factors.

**NP Physicochemical Properties**	Particle size distribution (ENMs in air and water, ambient particles in air and water)	
	Aqueous solubility	
	Reaction rate constant	
	Attachment factor (to ambient particles)	
	Density	
**Intermedia transport parameters**	*Process*	*Major factors*
	Dry Deposition	Temperature, wind speed, atmospheric stability, humidity, surface characteristics, ambient aerosols PSD
	Precipitation scavenging	Precipitation intensity, cloud base height, ambient aerosols PSD
	Aerosolization	Wind speed
	Soil wind resuspension	Wind speed, atmospheric stability, soil surface characteristics
	Soil runoff	Precipitation intensity, soil surface characteristics, ground incline degree
	Foliage washoff	Precipitation, foliage properties (e.g., water holding capacity), foliage coverage
	Sedimentation	PSD and density of suspended solids
	Sediment resuspension	Water bottom current velocity, sediment type and roughness, wind speed, depth of water body

ENM Lifecycle Mass Release Equations

ENM release rates to air, water, and soil are given as [1,2]:

$$M_A = \text{ } mass\ release\ rate\ to\ air$$
$$= M_{prod}\left[F_{m,a}\sum_i\left(F_{u,a,i}+F_{d,a,i}\right)+\ F_{d,I}\times T_{I,air}\ +\ F_t\times T_{t,b}\times T_{b,I}\times T_{I,air}\right] \qquad [2]$$

$$M_W = mass\ release\ rate\ to\ water$$
$$= M_{prod}\left[T_{t,e}\times\left(F_{m,t}+\sum_i\left(F_{u,t,i}+F_{d,t,i}\right)\right)+F_{u,w}\right] \qquad [3]$$

$$M_S = mass\ release\ rate\ to\ soil$$
$$= M_{prod}\left[\sum_i F_{u,s,i}\ +\ F_t\times T_{t,b}\times T_{b,s}\right] \qquad [4]$$

where M_{prod} is the total mass production rate, and *F* and *T* are transfer coefficients [1]. The lifecycle stages manufacturing, use, and disposal are represented by subscript *m*, *u*, and *d*, respectively. The technical compartments refers to waste incineration plant (WIP), wastewater treatment plant (WWTP), and biosolids, and are denoted by subscripts *I, t,* and *b*, respectively. The WWTP effluent is designated by subscript *e*. The environmental compartments air, water, and soil are denoted by *a, w, and s*. The subscript *i* represents various ENM applications (e.g., cosmetic, coating/paints/pigments, electronics/optics).

Details of Use Cases

Use case 1: Environmental ENM concentrations and mass distribution. The typical use case of the RedNano integrated simulation tool is to estimate environmental ENM concentrations and mass distributions based on a specified scenario as per the workflow described in the Graphical User Interface section (Figure 6). It is noted that the parameter input does not need to follow a specific order. Also, the scenario design is checked internally at the GUI level prior to execution to ensure that the scenario is properly conceived (e.g., parameter values are within reasonable constrains, source release or initial compartmental concentration are non-zero). The simulation results can then be explored via the data visualization modules accessible via GUI (Figure 1).

Use case 2: Dynamic response of environmental system to temporally varying ENM release rates. ENM release rates are recognized as one of the most important parameters in environmental multimedia assessment [3]. The case of a constant (i.e., time-invariant) release rate, for estimation of steady state concentrations in the various environmental media, is a commonly used scenario [2-5]. However, time-dependent release rates may also be of interest. For example, ENM releases from sunscreens to water bodies in coastal cities may follow a sinusoidal function, where the releases in the summer may be significantly higher than those in the winter. Similarly, releases of ENMs due to vehicular traffic (either from automobile exhaust or due to release of carbon from wear of tires) may follow a periodic function with release rates during the day being greater than night. Additionally, the time required for the environment to recover (i.e., for ENMs to be removed from the environment via various transport processes) after the cessation of source release (e.g., after incidental spill) may also be of interest. Accordingly, within RedNano, simulations can be carried out to evaluate ENM distributions with

different ENM release kinetics. The source release can be simulated as a single or repeating release events, and the release rate of the events can be either a constant rate or given by sinusoidal functions [3], where the cycle period, cycle gap (for repeating events), and amplitude (for sinusoidal releases) can be specified. The source release function takes the following functional form:

$$R(t) = \begin{cases} A \cdot \sin\left(t \cdot \frac{\tau}{\pi}\right) + r, & \left(t \bmod (\tau + g)\right) \le \tau \\ 0, & \left(t \bmod (\tau + g)\right) > \tau \end{cases} \quad [5]$$

where $R(t)$ is the ENM release rate (kg s^{-1}) at t^{th} day, A (kg s^{-1}) is the amplitude of the sinusoid [3], τ (day) is the cycle period, g (day) is the cycle gap period, and r (kg s^{-1}) is the average release rate.

Use case 3: Impact of specific intermedia transport processes on the temporal dynamics of ENM distribution in the environment. To examine the impact of intermedia transport on ENM environmental distribution and to assess the effect of specific intermedia transport processes individually, one can construct scenarios that consider selected intermedia transport process(es) independently from each other, and from source release. The above may be accomplished by setting a non-zero initial ENM media concentration and setting the source release rate to zero. Additionally, one may carry out a series of simulations with varying meteorological and geographical parameters, and thus varying intermedia transport rates, to evaluate the quantitative dependency of multimedia distribution on specific parameters. Examples demonstrating the above was provided in the main text for dry deposition and rain scavenging. An additional illustrative example is provided below for wind dilution.

ENMs can be removed from the modeled atmospheric airshed (to neighboring airsheds) by the outflowing wind, via the wind dilution process, which occurs when the ENM concentration in

the inflow wind is lower than that in the outflow wind. The rate of ENM removal by wind dilution is typically characterized by the convective residence time (or retention time) of the airshed, which is typically ≈10 h for an urban region such as Los Angeles. Under ideal conditions (i.e., with perfect mixing), the residence time (h) can be estimated via $\tau = V/Q$, where V (m^3) is the volume of the airshed, and Q ($m^3\ hr^{-1}$) is the volumetric flow of the wind [6]. However, flow recirculation and shortcuts in the region can cause non-ideal mixing, and can result in increases or decreases in the effective (or apparent) convective residence time [6]. In such case a correction factor, which may be obtained from tracer studies or determined via dispersion models, can be applied to correct the residence time [3]. The illustrative case of TiO_2 removal by wind dilution in Los Angeles is depicted in Figure S1, in which the time to remove 90% of ENMs from the airshed with convective residence time in the range of 5–20 h is ≈0.5–2 days, respectively. Although the time scale for ENM removal via wind dilution is typical longer than that of instantaneous rain scavenging removal of ENM from the atmospheric airshed, wind dilution may be more significant in removing ENM when averaged over long periods of time (e.g., years) due to the episodic nature of rain scavenging. For example, in Los Angeles, the mass of ENM removed in 1 yr via wind dilution is a factor of ≈27 greater than via rain scavenging (to vegetative canopy, soil, and water surfaces) (Figure S6).

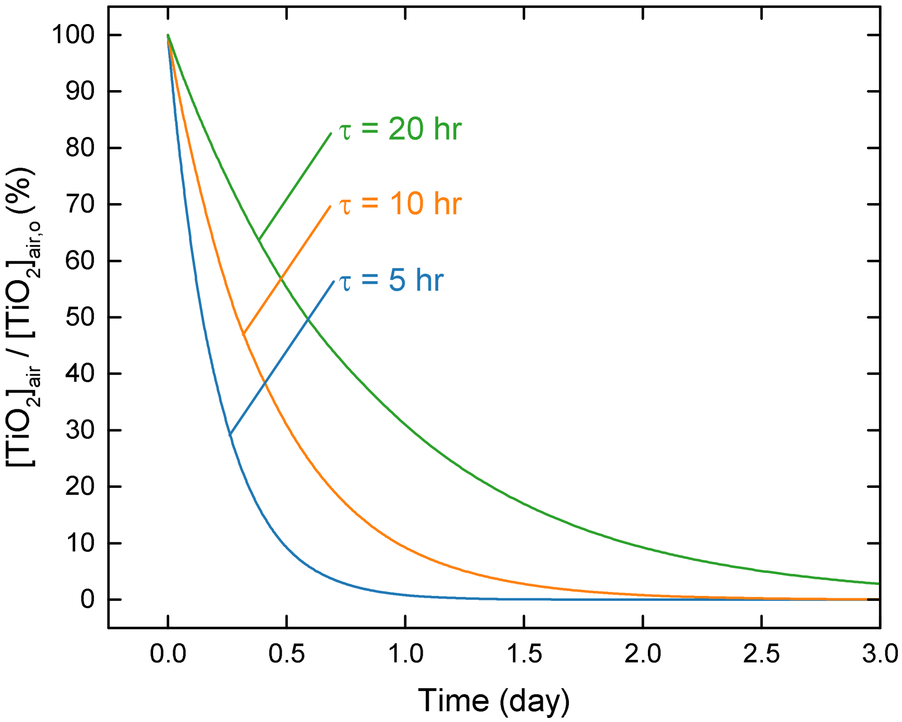

Figure S1: Effect of wind dilution on dynamics of TiO_2 concentration in air in Los Angeles as a function of convective residence time (τ) over the range of 5–20 h. TiO_2 concentration in air is reported as percent of its initial concentration, which is the predicted steady state concentration for TiO_2 in Los Angeles, and the source release is taken to be zero for all compartments. Regional geographical parameters are reported in Table S2.

Use case 4: Comparison of estimated environmental ENM concentrations in various regions. In order to evaluate the overall impact of ENMs on the environment, it is of interest to estimate the environmental distribution of ENMs in different regions (e.g., countries), by performing a series of simulations using geographical parameters, meteorological conditions, and source release rates specific to the regions under consideration. In this regard, it is noted that the parameter database in the present modeling platform contains a library of regionally specific geographical, meteorological parameters, and transfer coefficients for estimating ENM releases.

Use case 5: Contribution by application to ENM environmental distribution. Contribution of the application to ENM release and environmental distribution may provide useful information to researchers as well as assist the regulatory community, since ENMs may undergo transformation (e.g., surface functionalization) specific to an application [7] throughout their life cycle. The above can be accomplished with the present modeling platform, by estimating release rate of a given ENM associated with a specific application via LearNano, and evaluate the associated multimedia distribution with MendNano.

Use case 6: Estimation of source release rates, based on matching of model estimates and reported environmental concentrations. ENM release rates can be estimated by iteratively executing simulations with varying ENM release rates to match the measured ENM concentrations. Using a Newton–Raphson's iteration, one can achieve rapid matching between estimated and reported concentrations. This approach is useful, for example, for retrospective estimates of ENM release rates of ENMs. The above use case can also be utilized to check for consistency between reported ENM release rate, and measured ENM concentrations.

Estimation of Atmospheric CeO_2 Release Rates in Newcastle UK by VMT and Diesel Fuel Consumption

Estimated CeO_2 release rate based on vehicle miles travelled (VMT)

Since VMT for buses was not reported specifically for Newcastle, the estimated VMT for England [8] was used, and scaled to Newcastle on the basis of population ratio. The CeO_2 release rate to air was subsequently estimated using typical diesel bus fuel efficiency [9] and CeO_2 concentration [10] in the fuel additive.

Table S2: Parameters for estimating CeO_2 release rates.

Parameter	Value	Unit	Ref
Diesel bus fuel efficiency (*f*)	6.0	miles gal^{-1}	[9]
=	2.55	km L^{-1}	
VMT, England	1,298,000,000	miles yr^{-1}	[8]
Population, England	53.5	million people	[11]
Population, Newcastle, UK	280,200	people	[12]
CeO_2 concentration in diesel fuel additive	5	mg L^{-1}	[10]

$$Release = \left(\frac{280200}{53.5\times10^6}\right)\cdot 1298\times10^6\,\frac{mile}{yr}\cdot\left(1.609\,\frac{km}{mile}\right)\cdot\left(\frac{L}{2.55\,km}\right)\cdot\left(5\,\frac{mg}{L}\right)\cdot\left(\frac{kg}{10^6\,mg}\right)$$

$$= 21.48\,kg/yr$$

Estimated CeO_2 release rate based on fuel consumption

The release rate of CeO_2 from diesel fuel additive was also estimated based on reported fuel consumption data for a town (Northumberland) in the same region (Northeast UK) with similar population (316,028). Total fuel consumption by buses for the above city was reported to be 7.7

KTonne year^{-1}[13], which was then scaled to Newcastle on a population basis. The density of diesel fuel is taken to be 0.832 kg L^{-1}.

$$Release = \left(\frac{280200}{316028}\right)\cdot\left(7700\,\frac{tonne}{yr}\right)\cdot\left(1000\,\frac{kg}{tonne}\right)\cdot\left(\frac{L}{0.745}\right)\cdot\left(5\,\frac{mg}{L}\right)\cdot\left(\frac{kg}{10^6\,mg}\right)$$

$$= 45.82\ kg/yr$$

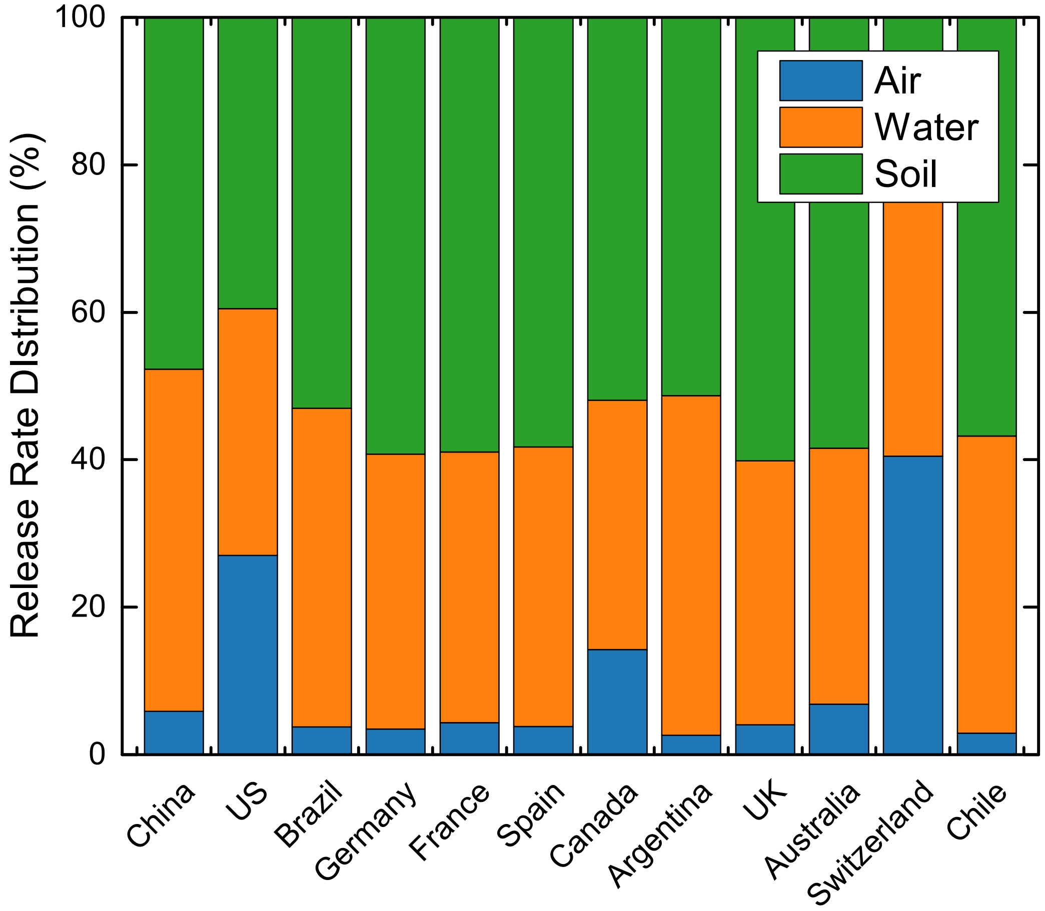

Figure S2: CeO_2 Release rate distribution (between air, water, and soil) for 12 selected countries. High estimate of the release rates are depicted.

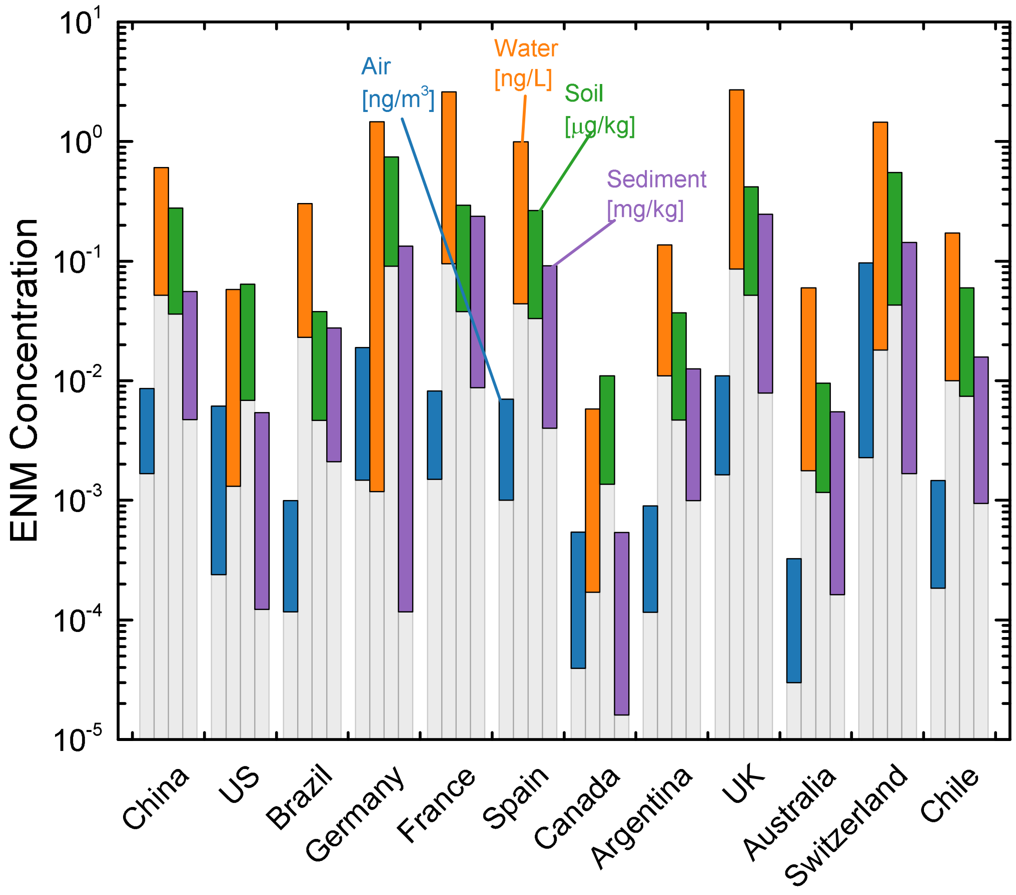

Figure S3: Estimated range of regional average CeO_2 compartmental concentrations for 12 selected countries at the end of 1-year simulation.

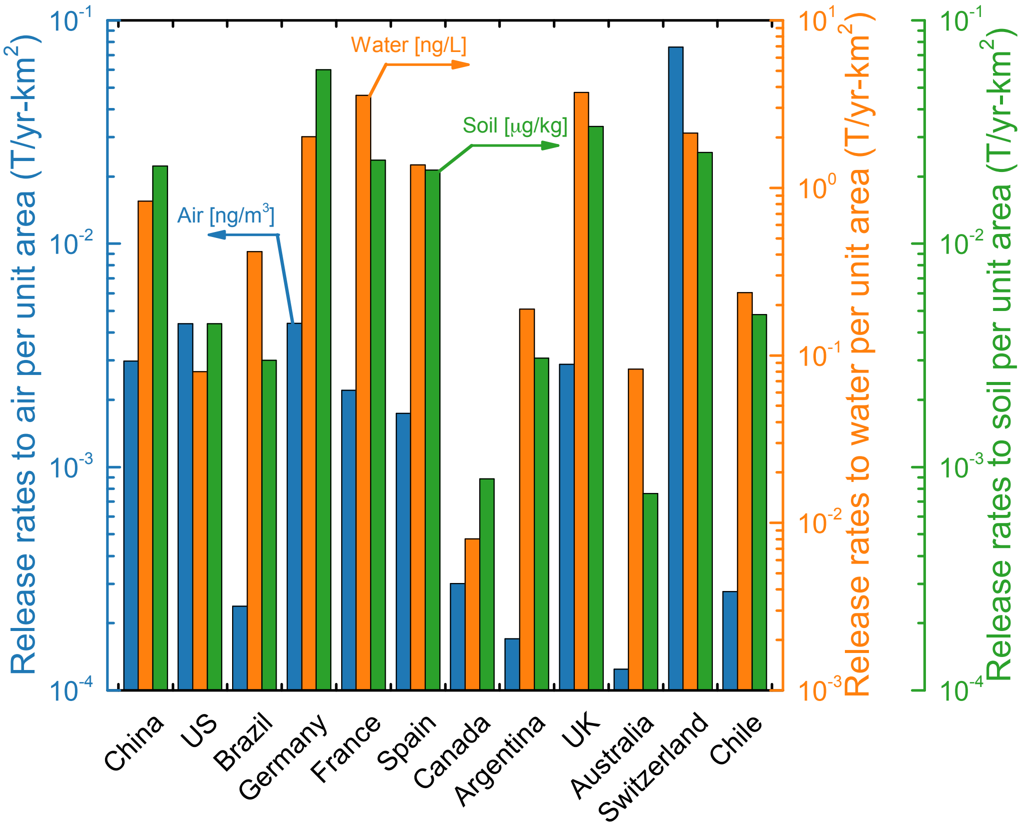

Figure S4: CeO_2 release rates (high estimate) per unit area for 12 selected countries. The air–soil and air–water interfacial areas are listed in Table S3.

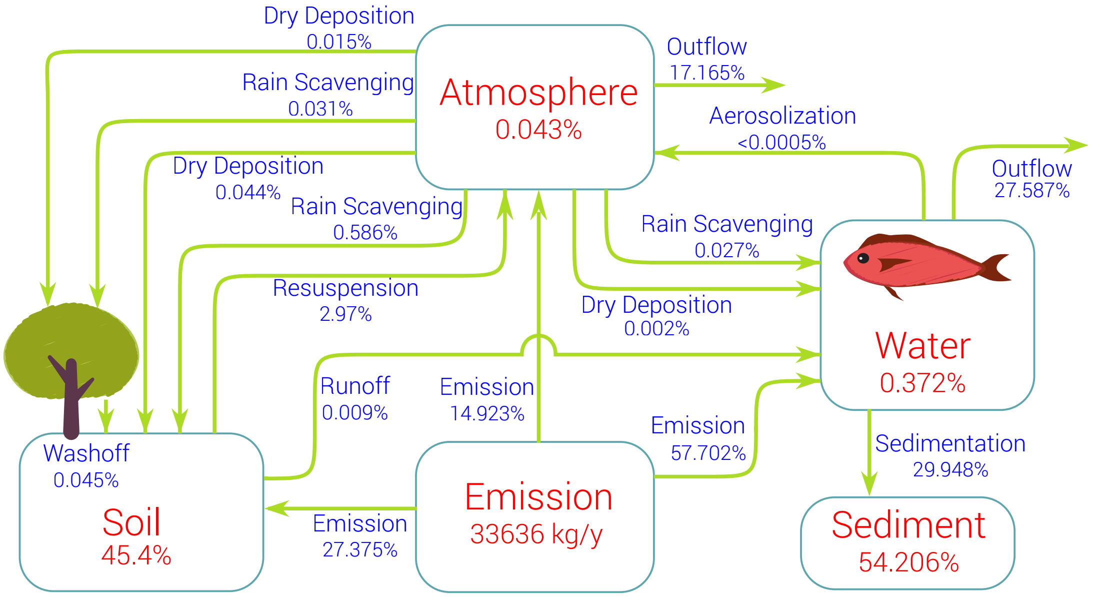

Figure S5: Intermedia transport rates of TiO_2 and mass distribution among the various compartments at the end of 1-year simulation for the Los Angeles test case. TiO_2 release rates are reported in Table S5, and regional geographical and meteorological parameters are reported in Table S4. Intermedia transport rates (in blue font) are reported as percent of total ENM release rate, and the mass distribution of ENM for each compartment is reported as percent of total ENM mass in the environment (in red font).

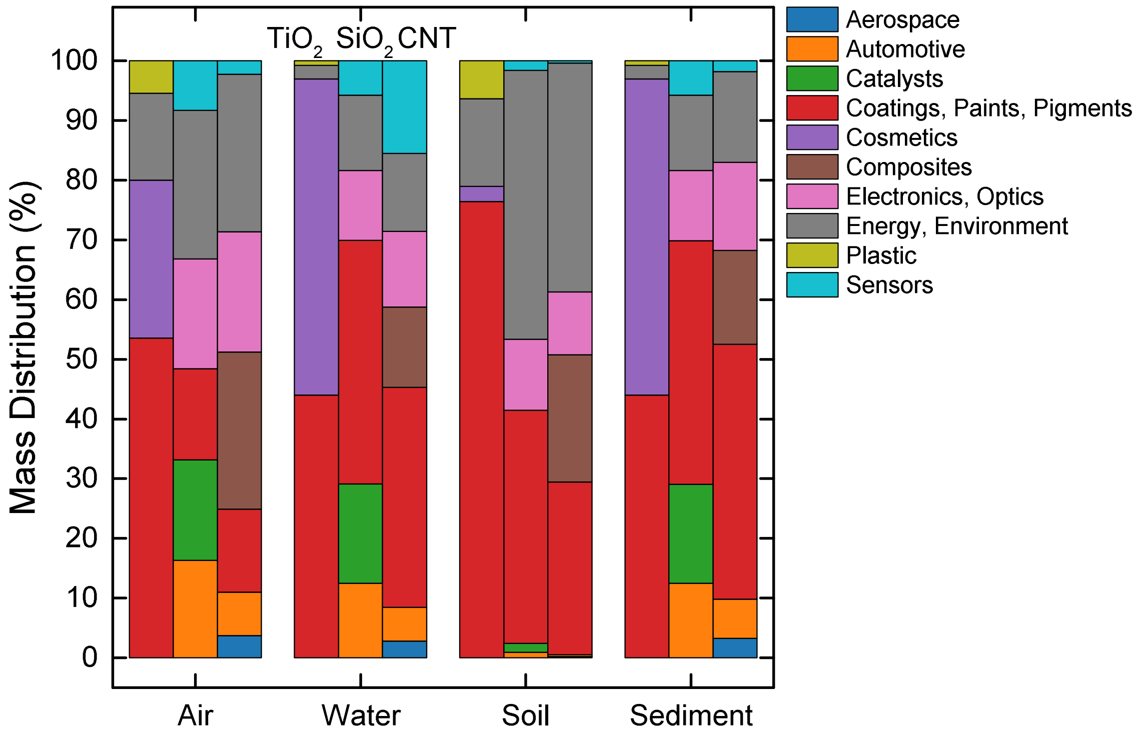

Figure S6: Contribution of various applications to the compartmental mass distribution of TiO_2, SiO_2, and CNT at the end of 1-year simulation for the Los Angeles test case. ENM release rates and regional geographical and meteorological parameters are reported in Table S5 and Table S4, respectively.

Table S3: Parameters for simulation of ENM distributions in various countries.

Country	Soil Area (km^2)[14]	Water Area (km^2) [14]	Annual rain fall (mm) [14]
Argentina	27,36,690	43,710	591
Australia	76,33,565	58,459	534
Brazil	84,60,415	55,352	1,782
Canada	90,93,507	891,163	537
Chile	7,43,812	12,290	1,522
China	93,26,410	270,550	645
France	6,40,427	3,374	867
Germany	3,48,672	8,350	700
Spain	4,98,980	6,390	636
Switzerland	39,997	1,280	1,537
UK	2,41,930	1,680	1,220
US	91,61,966	664,709	715

Common Parameter	Parameter Value	
Atmospheric mixing height[2]	1000	m
Depth of soil[2]	0.1	m
Depth of water[2]	3	m
Depth of sediment[2]	0.03	m
Average wind speed	3	$m\ s^{-1}$
Dry soil density[2]	1,500	$kg\ m^{-3}$
Dry sediment density[2]	260	$kg\ m^{-3}$
Ambient aerosol PSD (Table 8.3 in Seinfeld and Pandis)[15]	Rural	
Ambient aerosol density	1,500	$kg\ m^{-3}$
<u>Parameters of lognormal size distribution of Suspended Solids in water compartment</u>		
Mode	5	μm
μ_{ln}	8.5	nm
σ_{ln}	0.6	nm
Ambient suspended solids density	1,500	$kg\ m^{-3}$
Initial and inflow concentration of ENMs in air and water	0	$ng\ m^{-3}$
Attachment factor	1	

Table S4: Parameters for simulation of ENM distributions in Los Angeles.

Parameter	Parameter Value	
Air-soil interface area[16]	1,213	km^2
Air-water interface area[16]	52.7	km^2
Atmospheric mixing height	1,000	m
Depth of Soil	0.05	m
Depth of Water	4.9	m
Depth of Sediment	0.03	m
Atmospheric convective residence time	10	hr
Water convective residence time	65	hr
Annual rainfall rate[17]	326	$mm\ yr^{-1}$
Average wind speed[17]	2.7	$m\ s^{-1}$
Dry soil density	1,500	$kg\ m^{-3}$
Dry sediment density	260	$kg\ m^{-3}$
Ambient aerosol PSD (Table 8.3 in Seinfeld and Pandis)[15]	Urban	
Ambient aerosol density	1,500	$kg\ m^{-3}$
<u>Parameters of lognormal size distribution of suspended solids in water compartment</u>		
Mode	5	μm
μ_{ln}	8.5	nm
σ_{ln}	0.6	nm
Ambient suspended solids density	1,500	$kg\ m^{-3}$
Attachment factor	1	
Initial and inflow concentration of ENMs in air and water	0	$ng\ m^{-3}$
Foliage area per unit soil area (leaf area index)[18]	2.87	$m^2_{foliar}\ kg^{-1}_{plant}$
Fraction of soil covered by vegetation[19]	0.5	

Note: for the simulation results shown in Figures 10, 11, S2, the values for wind speed, rainfall rate, and convective residence time are reported in the figures.

Table S5: Release rates of TiO_2, SiO_2, and CNT in Los Angeles.

ENM and Application	Release [a] (kg yr^{-1})			
	Air	Water	Soil[b]	Soil[c]
TiO_2				
Coatings, Paints, Pigments	2249 (5.24%)	8528 (19.87%)	7100 (16.54%)	11248 (26.21%)
Cosmetics	1789 (4.17%)	10293 (23.98%)	167 (0.39%)	5107 (11.9%)
Energy, Environment	729 (1.7%)	438 (1.02%)	1352 (3.15%)	1499 (3.49%)
Plastic	253 (0.59%)	149 (0.35%)	589 (1.37%)	639 (1.49%)
SiO_2				
Automotive	947 (5.85%)	625 (3.86%)	5 (0.03%)	213 (1.31%)
Catalysts	971 (5.99%)	833 (5.15%)	27 (0.17%)	355 (2.19%)
Coatings, Paints, Pigments	539 (3.33%)	2046 (12.64%)	1703 (10.52%)	2698 (16.67%)
Electronics, Optics	968 (5.98%)	587 (3.63%)	487 (3.01%)	672 (4.15%)
Energy, Environment	1050 (6.48%)	631 (3.89%)	1947 (12.02%)	2157 (13.32%)
Sensors	470 (2.91%)	288 (1.78%)	51 (0.32%)	141 (0.87%)
CNT				
Aerospace	8 (1.34%)	5.3 (0.89%)	0.05 (0.01%)	1.8 (0.3%)
Automotive	15.9 (2.69%)	10.5 (1.77%)	0.1 (0.02%)	3.6 (0.6%)
Coatings, Paints, Pigments	18.2 (3.06%)	68.9 (11.61%)	57.4 (9.67%)	90.9 (15.32%)
Composites	48.1 (8.1%)	25.3 (4.27%)	41 (6.91%)	49.1 (8.28%)
Electronics, Optics	39.1 (6.59%)	23.7 (4%)	19.7 (3.32%)	27.2 (4.58%)
Energy, Environment	40.7 (6.85%)	24.4 (4.12%)	75.4 (12.71%)	83.6 (14.08%)
Sensors	4.8 (0.8%)	2.9 (0.49%)	0.5 (0.09%)	1.4 (0.24%)

[a] Values in parentheses represent the indicated release rates (outside of the parentheses) as percent of total release rate to the environmental compartments for the specified ENM (i.e., release to air, water, and soil).
[b] Direct release to soil compartment (i.e., not including release from WWTP)
[c] Total release to soil compartment via the sum of direct release and release associated with WWTP sludge

References

1. Keller, A.; McFerran, S.; Lazareva, A. Suh, S. *Journal of Nanoparticle Research* **2013**. 15(6), 1-17. doi: 10.1007/s11051-013-1692-4
2. Gottschalk, F.; Sonderer, T.; Scholz, R.W. Nowack, B. *Environmental Science & Technology* **2009**. 43(24), 9216-9222. doi: 10.1021/es9015553
3. Liu, H.H. Cohen, Y. *Environmental Science & Technology* **2014**. 48(6), 3281-3292. doi: 10.1021/es405132z
4. Praetorius, A.; Scheringer, M. Hungerbuhler, K. *Environmental Science & Technology* **2012**. 46(12), 6705-6713. doi: 10.1021/es204530n
5. Meesters, J.A.J.; Koelmans, A.A.; Quik, J.T.K.; Hendriks, A.J. van de Meentt, D. *Environmental Science & Technology* **2014**. 48(10), 5726-5736. doi: 10.1021/es500548h
6. Cohen, Y. Ryan, P.A. *Environmental Science & Technology* **1985**. 19(5), 412-417. doi: 10.1021/es00135a004
7. Dale, A.L.; Lowry, G.V. Casman, E.A. *Environmental Science & Technology* **2013**. 47(22), 12920-12928. doi: 10.1021/es402341t
8. Tranter, M., *Annual Bus Statistics: England 2012/13*, U.D.f. Transport, **2013**,
9. Norton, P., *Demonstration of Caterpillar C-10 Dual-Fuel Engines in MCI 102DL3 Commuter Buses*, **2000**, Natinoal Renewable Energy Laboratory: Golden, CO.
10. Park, B.; Donaldson, K.; Duffin, R.; Tran, L.; Kelly, F.; Mudway, I.; Morin, J.-P.; Guest, R.; Jenkinson, P.; Samaras, Z.; Giannouli, M.; Kouridis, H. Martin, P. *Inhalation Toxicology* **2008**. 20(6), 547-566. doi: 10.1080/08958370801915309
11. Office for National Statistics, *Population Estimates for UK, England and Wales, Scotland and Northern Ireland, Mid-2011 and Mid-2012*, **2013**.
12. Office for National Statistics, *Census result shows increase in population of the North East*, **2012**.
13. Khan, S., *Sub-national road transport fuel consumption statistics*, UK Department of Energy & Climate Change, **2012**,
14. The World Factbook, in **2013**
15. Seinfeld, J.H. Pandis, S.N., *Atmospheric chemistry and physics : from air pollution to climate change*. 2nd ed.; J. Wiley: Hoboken, N.J. **2006**.
16. USCB. United States Census Bureau QuickFacts, in **2012**
17. National Climatic Data Center, in **2013**
18. Breuer, L.; Eckhardt, K. Frede, H.-G. *Ecological Modelling* **2003**. 169(2–3), 237-293. doi: 10.1016/s0304-3800(03)00274-6
19. Clay, E.R., *Multimedia Environmental Distribution of Gaseous, Dissolved, and Particle-bound Pollutants*, in *Department of Chemical Engineering***1992**, University of California, Los Angeles: Los Angeles. p. 302.
